# The Korean Medicine HOme Medical care for the Elderly (K-HOME) registry: A study protocol for a multicenter registry on aging in place and functional recovery

**DOI:** 10.1371/journal.pone.0347574

**Published:** 2026-04-30

**Authors:** Hanbit Jin, Yerin Bae, Soomin Song, Hyungsun Jun, Eunji Ahn, Jieun Yu, Beumseuk Kim, Dongsu Kim

**Affiliations:** 1 Department of Preventive Medicine, College of Korean Medicine, Dongshin University, Naju, Republic of Korea; 2 Department of Diagnostics, College of Korean Medicine, Dongshin University, Naju, Republic of Korea; 3 Department of Humanities and Social Medicine, The School of Korean Medicine, Pusan National University, Yangsan, Republic of Korea; 4 Jungdong Korean Medicine Clinic, Bucheon, Republic of Korea; Health Sciences University Istanbul Training and Research Hospital, TÜRKIYE

## Abstract

**Objective:**

This study aims to evaluate 1-year changes in functional status and aging-in-place (AIP)–relevant outcomes among homebound older adults receiving care through Korean Medicine home medical centers (KM-HMCs). The primary objective is to evaluate changes in activities of daily living measured by the Korean Activities of Daily Living (K-ADL) scale. Secondary objectives are to track major events (mortality, long-term hospitalization, and admission to long-term care facilities) and to examine factors associated with response.

**Materials and methods:**

This multicenter, prospective, registry-based observational study will be conducted at six KM-HMCs in South Korea. We aim to enroll approximately 250 participants aged 65 years or older with functional decline to obtain an analytic sample of about 200 after expected attrition. Participants will receive usual interprofessional home-based services. The primary outcome is the change in K-ADL from baseline to 1 year. Secondary outcomes include frailty assessed by the Korean version of the FRAIL scale, pain intensity assessed by a numerical rating scale, and AIP-related events. Analyses will use paired tests for changes over 1 year and multivariable logistic regression for factors associated with response, which was operationally defined as a decrease of ≥1 point in the K-ADL score from baseline to 1 year.

**Discussion:**

This registry will provide standardized longitudinal data on functional status and key events in a routine-care setting, addressing evidence gaps in Korean Medicine home medical care for AIP. Findings may inform evaluation and refinement of the KM-HMC model and guide future research and policy discussions.

## Introduction

South Korea is undergoing a rapid demographic transition toward an aged society [1]. In 2017, the proportion of people aged 65 years or older surpassed 14% of the total population, and by 2025 it exceeded 20%, marking Korea#39;s entry into a super-aged society [2]. Population aging has increased the burden of chronic illness and geriatric syndromes, while healthcare delivery remains largely oriented toward hospitals and long-term care facilities, limiting access to medical care for homebound older adults [3,4]. Given that long-term care services have historically emphasized institutional care and basic nursing support, frail older adults with mobility limitations often face barriers to timely and continuous medical care in the community [4].

Home-Based Primary Care (HBPC) supports longitudinal management of chronic conditions in patients’ living environments. Indeed, systematic reviews of HBPC programs for homebound older adults indicate associations with reduced hospital and emergency department use, lower healthcare costs, and high patient and caregiver satisfaction [5]. Concurrently, Aging in Place (AIP)—supporting older adults to remain at home while receiving appropriate medical and social support—has become a key policy objective [6]. In response, the South Korean government launched the Long-Term Care Home Medical Center (LTC-HMC) pilot program in late 2022. Under this program, designated home medical centers provide integrated HBPC for immobile or frail older adults enrolled in long-term care through interprofessional teams that typically include a physician or a licensed Korean Medicine doctor (KMD), a nurse, and a social worker. In this study, LTC-HMCs operated by Korean Medicine (KM) clinics are referred to as Korean Medicine home medical centers (KM-HMCs), where KMDs provide clinical care and participate in team-based service delivery, including KM interventions in home-based settings.

Despite the expansion of pilot services and early reports of favorable indicators [7], HBPC in Korea remains at an early stage of implementation, and evidence on longer-term outcomes and cost-effectiveness remains scarce. Furthermore, existing evaluations often lack longitudinal data on functional status, institutionalization, and quality of life [8]. Functional status, commonly assessed using activities of daily living (ADL), is a core outcome reflecting independence and care dependency [9]; yet, longitudinal evidence on ADL trajectories among frail older adults receiving KM-led HBPC is limited.

Therefore, we established a multicenter registry of KM clinics designated as LTC-HMCs and participating in the national pilot project. The primary aim is to evaluate 1-year change in ADL among homebound older adults with functional decline receiving HBPC from KM-HMCs. Secondary aims are to track AIP-relevant events (long-term hospitalization, admission to long-term care facilities, and mortality) and to explore patient- and service-related factors associated with these outcomes.

## Materials and methods

### Registry aims

The primary objective is to evaluate 1-year change in ADL among older adults with functional decline receiving care through KM-HMCs, measured using K-ADL. Secondary objectives are to: (1) track AIP-relevant events (long-term hospitalization, admission to long-term care facilities, and mortality); (2) assess 1-year change in frailty and chronic pain using the Korean version of the FRAIL scale (K-FRAIL) and the Numerical Rating Scale (NRS); (3) examine patient- and service-related factors associated with outcomes; (4) describe participant characteristics and services delivered; and (5) build a standardized longitudinal database to support ongoing evaluation and future research.

### Study design and setting

This is a multicenter, registry-based observational study collecting standardized baseline and follow-up data in routine practice; no study-mandated interventions or treatment restrictions will be applied. The protocol was prepared with reference to the SPIRIT (Standard Protocol Items: Recommendations for Interventional Trials) 2025 guidance, where applicable (S1 File. SPIRIT 2025 checklist).

The registry will be implemented at six KM-HMCs (Jungdong, Healthy Village, Dongbang Shintong Bubu, Haemalgeun, Kim Jeong-cheol, and Seohwa) participating in the national pilot project, selected to capture geographic variation. Under the pilot project, sites provide interprofessional home-based services that typically include at least monthly visits by KM doctors, twice-monthly nurse visits, and periodic social worker counseling.

KM-HMC services encompass comprehensive home-based primary care delivered in collaboration with community resources. In addition to Korean Medicine interventions (e.g., acupuncture, moxibustion, herbal medicine), services include a broad range of routine medical and nursing care such as wound care, catheter management, medication support, and monitoring of chronic conditions. Care is provided through coordination with local healthcare providers, nurses, and social welfare services, and may involve referral and linkage to community-based resources as needed. The specific components and frequency of care are individualized based on clinical judgment and patient needs.

### Participants and eligibility

Participants will be recruited during routine home visits. Written informed consent will be obtained from all eligible patients or their legal representatives prior to enrollment.

Eligibility criteria are age ≥ 65 years, current receipt of services at a participating KM-HMC, and Long-Term Care Insurance grade 3 or 4; grades 1–2 are not enrolled to focus on participants for whom 1-year changes in K-ADL can be feasibly assessed and interpreted in this registry.

Exclusion criteria are expected survival of <3 months or anticipated inability to complete 1-year follow-up, concurrent participation in other studies that may affect outcomes, acute conditions requiring immediate hospitalization, or severe cognitive or communication impairment precluding assessments even with assistance. Proxy responses will be permitted when direct responses are not possible; proxies will be limited to legal representatives or primary caregivers providing direct care for an average of ≥3 hours/day [10,11].

### Patient and public involvement

Patients and the public were not involved in the design, conduct, reporting, or dissemination plans of this study. Patient perspectives will be assessed using the Client Satisfaction Questionnaire (CSQ).

### Sample size

Because this is a registry-based observational study, the target sample size was set based on feasibility and the planned secondary analyses. Across the six participating sites, approximately 370 patients are expected to meet eligibility criteria. Assuming a participation rate of 70%, the registry aims to enroll approximately 250 participants. Allowing for 20% attrition due to mortality or institutionalization, the expected final analytic sample is approximately 200 participants.

For logistic regression of response, we prespecified response as a decrease of ≥1 point in the K-ADL score from baseline to 1 year. Because a validated minimal clinically important difference (MCID) for the K-ADL has not been established, this threshold was selected pragmatically, informed by indirect evidence from related basic ADL measures [12,13]. Based on this definition, we assumed a response rate of 35–45% according to a previous rehabilitation trial reporting recovery of ADL performance at 12 months [14]. With an expected final analytic sample of 200 participants, this would yield approximately 70–90 responder events, which is sufficient to support up to eight prespecified covariates based on an events-per-variable ratio of approximately 10 [15,16]. The prespecified covariates are age, sex, comorbidity count, baseline K-FRAIL, baseline K-ADL, Five Times Sit-to-Stand Test (FTSST), NRS, and service utilization, selected based on clinical relevance and prior evidence [17–20].

### Study procedures

**Fig 1:**
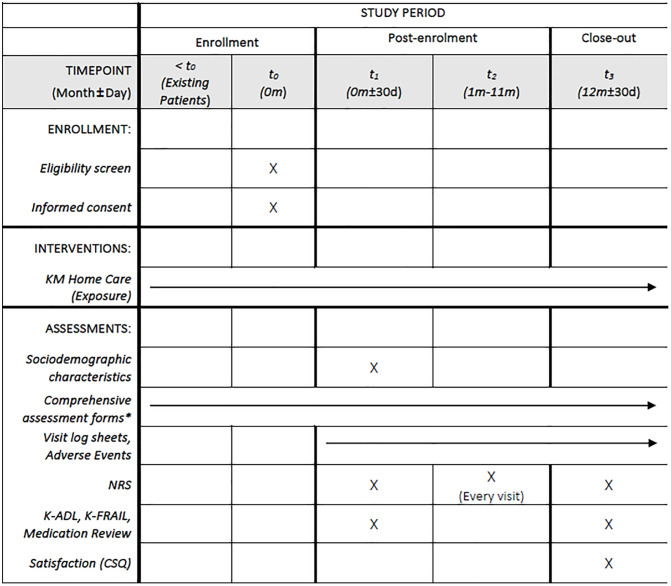
SPIRIT schedule. * The official evaluation form mandated by the pilot project, updated annually. Data are collected prospectively for new patients and retrospectively for existing patients to retrieve initial baseline data.

The study timeline includes enrollment, baseline assessment, monitoring for 1 year, and a 1-year follow-up assessment (Fig 1). For new patients, baseline data are collected prospectively in the Case Report Form (CRF) at enrollment. For existing patients, the initial Comprehensive Assessment and Care Plan established at the commencement of home care services defines the start of exposure to the KM-HMC intervention; their current clinical status at enrollment is also recorded prospectively in the CRF. Throughout the study period, usual services will continue. Administrative records, including Comprehensive Assessments and Care Plans, and visit logs, are completed as part of routine practice; data from these records will be collected prospectively for new patients, while for existing patients, historical data will also be collected.

### Outcomes

The primary outcome is the change in K-ADL from baseline to 1 year. K-ADL is a 7-item measure (score range 7–21), where lower scores indicate greater independence [18]. Secondary outcomes assessed at the 1-year interval include frailty status according to K-FRAIL (0: robust, 1–2: pre-frail, 3–5: frail) [17], changes in medication use (polypharmacy and deprescribing patterns), and patient satisfaction measured once at 1 year using the CSQ [21]. AIP indicators (mortality, long-term hospitalization >30 days, and admission to long-term care facilities) are recorded upon occurrence [22–24], while chronic pain intensity measured by the NRS (0–10) is assessed at every visit. Additional outcomes and covariates will be derived from pilot program–mandated administrative records, including the Comprehensive Assessment and Care Plan and visit logs, which capture sociodemographic characteristics, health and functional status, and service delivery details.

### Data collection and management

#### Data collection and quality control.

Data entry follows a two-step process: (1) completed paper CRFs and de-identified copies of relevant administrative records (study ID only) will be securely transferred to the Central Data Management Team at Dongshin University; and (2) the central team will check for completeness and consistency and enter the data into a web-based electronic CRF system (iClick, managed by the National Institute for Korean Medicine Development).

To reduce inter-rater variability and maintain consistency across sites, all evaluators will complete standardized training before study initiation, including standard operating procedures for K-ADL, K-FRAIL, and other assessments. Inter-rater reliability will be assessed early in the study. Data monitoring will be conducted by an independent, monitoring-trained KM doctor via on-site visits, which will include a comprehensive review of all source documents. To minimize attrition, staff will build rapport during regular visits and conduct prompt telephone follow-up after missed appointments to reschedule visits and address barriers to continued participation.

### Safety considerations

As an observational registry without experimental interventions, study-related risk is expected to be minimal. Mortality, long-term hospitalization (>30 days), and admission to a long-term care facility will be tracked as key events relevant to safety and sustainability. If such an event occurs, follow-up will be discontinued and the reason recorded in the CRF. Given the minimal-risk nature of the study, a formal data monitoring committee will not be convened; safety oversight will be conducted through regular investigator meetings and the independent monitoring process described above.

### Statistical analysis

Analyses will be performed in R (version 4.5.2) using RStudio (version 2025.09.2). All tests will be two-sided with α = 0.05. Baseline characteristics will be summarized using descriptive statistics.

The primary outcome (change in K-ADL from baseline to one year) will be evaluated in the overall cohort using a paired t-test or Wilcoxon signed-rank test, depending on distributional assumptions. Because the registry includes both newly enrolled and pre-existing patients, baseline functional status and prior exposure to KM-HMC services may differ between participants. Baseline characteristics, including baseline K-ADL, will therefore be compared between newly enrolled and pre-existing patients. To assess the robustness of the findings in the presence of this mixed cohort structure, a sensitivity analysis will be performed using multivariable linear regression with change in K-ADL as the dependent variable, adjusting for baseline K-ADL, months of prior service exposure, and registration status (new vs. existing). No interim analysis is planned.

Longitudinal NRS (measured at each visit) will be analyzed using a linear mixed-effects model with a random intercept and time treated as a continuous variable. Other secondary continuous outcomes (e.g., K-FRAIL) will be analyzed using paired t-tests or appropriate non-parametric alternatives.

Factors associated with response will be examined using multivariable logistic regression, with response operationally defined as a decrease of ≥1 point in the K-ADL score from baseline to 1 year. Participants who die or are permanently institutionalized during follow-up will be classified as non-responders to reduce attrition-related bias. Prespecified covariates are age, sex, comorbidity count, baseline K-FRAIL, baseline K-ADL, FTSST, baseline NRS, and service utilization frequency. Model fit, multicollinearity, and influential observations will be assessed using standard diagnostics.

Missing data will be handled using multiple imputation by chained equations (MICE), creating 20 imputed datasets and including auxiliary variables related to health status and service use; estimates will be pooled using Rubin’s rules. Exploratory analyses using administrative data (e.g., service utilization patterns and detailed assessments) will be conducted for hypothesis generation, with methods selected according to the research question and data structure.

### Ethics and dissemination

This protocol was approved by the Public Institutional Review Board (IRB) designated by the Ministry of Health and Welfare (No. P01-202510-01-034; October 21, 2025) and will be conducted in accordance with the Declaration of Helsinki. The protocol is also registered with the Clinical Research Information Service (CRIS) (No. KCT0011283; Protocol Version 1.4, December 9, 2025; URL: https://cris.nih.go.kr/cris/search/detailSearch.do?seq=31625&search_page=L). The full study protocol is provided in the Supplementary Materials (**S2** and S3 Files. Study protocol for IRB).

Written informed consent will be obtained from all participants; for those unable to consent, consent will be obtained from legal representatives. Participants will be asked to provide optional consent for sharing de-identified data with NIKOM for secondary analyses; refusal will not affect participation. All data will be de-identified using unique study codes. The re-identification key will be stored separately at each site in a locked cabinet or password-protected computer, accessible only to authorized investigators. As described in the informed consent form, de-identified individual participant data will be shared with the National Institute for Korean Medicine Development, retained for up to 10 years, and may be linked with public databases such as the National Health Insurance Service database for secondary analyses to inform policy development. Findings will be disseminated through peer-reviewed publications.

### Status and timeline

The study is currently recruiting. Participant enrollment began on November 5, 2025, and will continue until October 20, 2026. Data collection is scheduled to conclude by October 20, 2027.

## Discussion

This paper describes the protocol for the K-HOME registry, a multicenter prospective registry established within the LTC-HMC pilot project in South Korea. The registry aims to evaluate longitudinal changes in ADL and AIP-relevant outcomes among homebound older adults receiving services through KM-HMCs.

The registry is designed to address evidence gaps in KM home care. Compared with prior reports that were largely small in scale [25–27], this study will collect standardized longitudinal data on functional status and key events such as institutionalization and mortality over 1 year. By capturing routine practice, the registry may complement evidence from controlled trials and support evaluation of real-world service delivery across diverse patient profiles and practice patterns [28–30]. The registry will also describe the delivery of KM interventions and interprofessional team-based care within the participating sites.

Several limitations should be considered. First, the observational design without a control group limits causal inference. To mitigate confounding, analyses will adjust for prespecified covariates using multivariable models. Second, although sites were selected to capture geographic variation, participating centers and patients may not represent all home care users nationwide; standardized procedures and centralized data management are intended to improve consistency and reduce measurement bias. Third, participants are restricted to those with Long-Term Care Insurance grades 3–4, which may limit the generalizability of the findings to individuals with milder or more severe functional impairment.

The K-HOME registry provides an initial framework for longitudinal evaluation of KM home care in the LTC-HMC context. Future extensions may include broader eligibility (e.g., Long-Term Care Insurance grades 1–2) and additional measures appropriate for severe functional impairment and end-of-life care, as well as expansion of participating sites. Over time, registry data may inform the refinement of service delivery and contribute to real-world evidence to support standardization and reimbursement policy discussions in a super-aged society.

## Supporting information

S1 File SPIRIT 2025 checklist. (DOCX)

S2 File Study protocol for IRB v1.4_Kor (Effective Date: 2025. 11. 26). (DOCX)

S3 File Study protocol for IRB v1.4_Eng (Effective Date: 2025. 11. 26). (DOCX)
