# Supplementary material for: The Korean Medicine HOme Medical care for the Elderly (K-HOME) registry: A study protocol for a multicenter registry on aging in place and functional recovery
Source: PLoS One. 2026 Apr 30;21(4):e0347574. doi: 10.1371/journal.pone.0347574 (PMC13132427; doi:10.1371/journal.pone.0347574)
Supplement: S2 File — (DOCX) [file pone.0347574.s002.docx]

**[권고서식 제38호] 연구계획서(인간대상연구 실험연구용) ver4.0**

| ***보건복지부 지정 공용기관생명윤리위원회*** |
| --- |

**연구계획서**

**(인간대상연구 실험연구용)**

ver1.4

연구제목: 재택의료센터의 기능저하 노인을 대상으로 한 환자등록연구

Patient Registry Study of Elderly Patients with Functional Decline at Home Medical Centers

연구자 소속기관명 및 연구자명

중동한의원 김범석

건강한마을한의원 김권희

동방신통부부한의원 방호열

해맑은한의원 김창훈

김정철한의원 김정철

서화한의원 심희준

동신대학교 한의과대학 조교수 김동수

동신대학교 한의과대학 대학원생 진한빛

동신대학교 한의과대학 대학원생 안은지

동신대학교 한의과대학 연구원 배예린

원광대학교 한의과대학 대학원생 전형선

청년한의사회 송수민

**▣ 임상연구 계획서 제·개정 이력**

| **No** | **Version No.** | **Version Date** | **주요 내용** |
| --- | --- | --- | --- |
| 1 | 1.0 | 2023. 05. 20. | 임상연구 계획서 최초 제정 |
| 2 | 1.1 | 2023. 08. 04. | IRB 제출전 타 기관 수정사항 반영하여Ver 1.1로 수정 |
| 3 | 1.2 | 2025. 09. 11. | IRB 제출전 타 기관 수정사항 반영하여Ver 1.2로 수정 |
| 4 | 1.3 | 2025. 10. 13 | 공용IRB 심의의견 반영하여Ver 1.3으로 수정 및 IRB승인 |
| 5 | 1.4 | 2025. 11. 12 | 타 기관 수정사항 반영하여Ver 1.4로 계획 변경 및 IRB 승인 |

**1. 연구 배경**

**1) 국내외 연구개발과제 현황**

가. 사회경제적 배경

○ 국내 인구는 고령화 심화에 따라 노인의 기대수명 및 노년기 유병기간이 증가하고 있음.

- 우리나라 65세 이상 고령인구 비중은 2024년 기준 19.2%이고 2025년에는 20.3%가 되어 초고령사회에 진입할 것으로 예상됨.

**<그림** 1**> 국내 인구 기대수명 및 건강수명 추이**

출처: e-나라지표.(2023.05.10.). 기대수명(0세 기대여명) 및 유병기간 제외 기대수명(건강수명).
[https://www.index.go.kr/unity/potal/main/EachDtlPageDetail.do?idx_cd=2758](https://www.index.go.kr/unity/potal/main/EachDtlPageDetail.do\?idx_cd=2758)


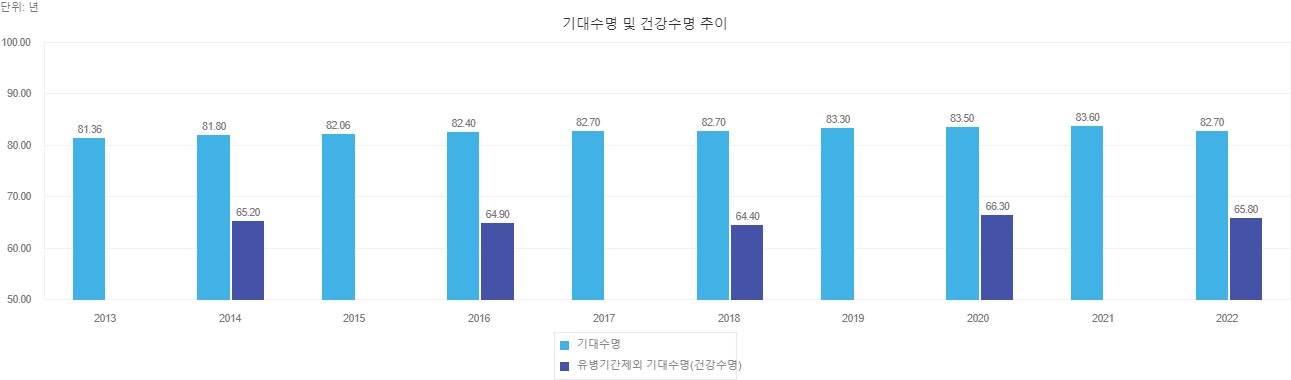


○ 65세 이상 노인층이 한의 진료비에서 차지하는 비중이 2014년 32%에서 2021년 37%로 증가함.

**<그림** 2**> 연도별 65세 기준 한의건강보험 진료비 추계**
출처: 국민건강보험공단, 건강보험심사평가원 (2014-2021)


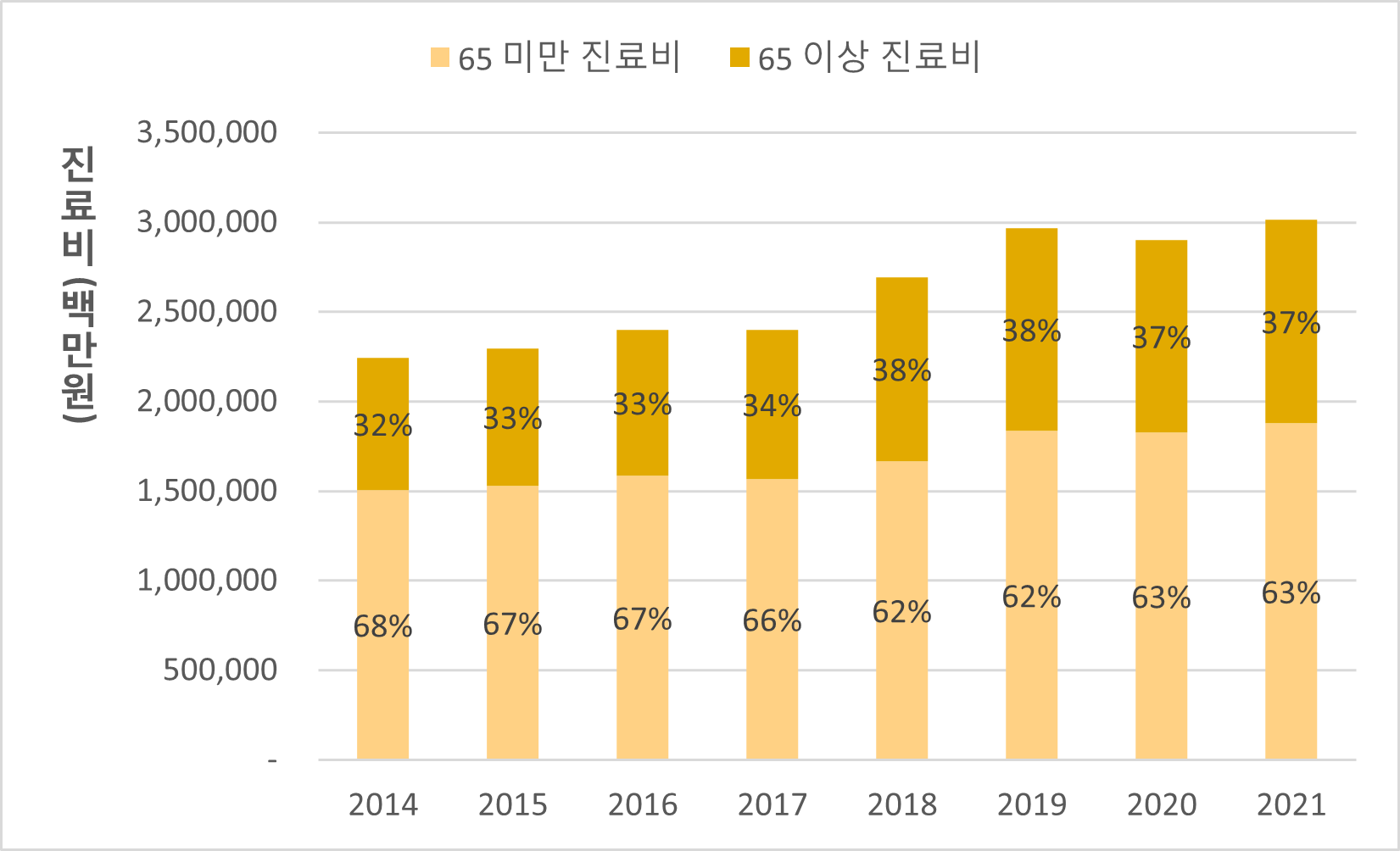


○ 정부는 노인에 대한 관리 수요 충족과 의료 재정 부담 완화를 위해 노인을 대상으로 포괄적인 관리 정책을 마련함.

- 2018년 정부는 지역사회 통합돌봄(커뮤니티 케어) 기본계획을 발표하고 2019년 6월부터 2년간 16개 시군구에서 ‘지역사회 통합돌봄 선도사업’을 시행함.
보건의료, 요양, 돌봄, 독립생활 등을 통합적으로 지원하는 지역주도형 사회서비스정책

- 의료·돌봄 수요 증가에 대응하고자 노인이 살던 곳에서 의료·돌봄 등의 서비스를 통합·연계적으로 지원받을 수 있는 ‘노인 의료·돌봄 통합지원 시범사업(＇23. 7.~＇25. 12.)’ 을 시행 중임.

- 장기요양 재택의료센터 시범사업은 장기요양 1~4등급 수급자 중 거동이 불편하여 재택의료가 필요하다고 의사가 판단한 자를 대상으로 하며, 의사 간호사 사회복지사 등이 주기적으로 가정을 방문하여 방문진료, 간호 및 지역사회 돌봄 연계 등을 제공함.

나. 기능 저하 노인의 정의와 현황

○ 정부는 2008년부터 기능 저하로 인하여 장기요양이 필요한 노인에게 요양에 대한 보장을 해주는 노인 장기요양보험제도(이하 장기요양보험)를 시행하고 있음.

- 장기요양보험 수급자는 노인성 질병을 가지고 있으며 신체적·인지적 기능이 저하로 인하여 일상생활을 혼자서 수행하기 어려운 자(6개월 이상의 기간동안)로 대표적인 기능저하 노인군으로 볼 수 있음.

| **<장기요양보험 대상자 정의>**  ∙ 65세 이상의 노인 또는 65세 미만의 자로서 치매ㆍ뇌혈관성 질환 등 대통령령으로 정하는 노인성 질병을 가진 자  ∙ 고령이나 노인성 질병 등의 사유로 일상생활을 혼자서 수행하기 어려운 노인  ∙ 장기요양보험제도는 이들에게 신체활동 또는 가사 활동 지원 등의 장기요양급여를 제공함  출처: 「노인장기요양보험법 제2조」의 내용 재구성 |
| --- |

- 수급자는 심신의 기능 상태에 따라 1~5등급 및 인지지원등급으로 판정되며 1~2등급은, 3~4등급은 5등급과 인지지원등급은 치매(「노인장기요양보험법 시행령」 제2조에 따른 노인성 질병에 해당하는 치매로 한정) 질환을 보유함.

**<표** 3**> 장기요양 등급 판정 현황**

| 구분 | | 계 | 인정자 | | | | | | | 등급외자 | | | |
| --- | --- | --- | --- | --- | --- | --- | --- | --- | --- | --- | --- | --- | --- |
|  |  |  | 소계 | 1등급 | 2등급 | 3등급 | 4등급 | 5등급 | 인지 지원  등급 | 소계 | 등급외A | 등급외B | 등급외C |
| 2024년  (3분기) | 인원 | 1,282,878 | 1,147,469 | 54,519 | 98,989 | 307,216 | 527,096 | 132,393 | 27,256 | 135,409 | 73,372 | 43,758 | 18,279 |
|  | 비율 | 100% | 89.40% | 4.20% | 7.70% | 23.90% | 41.10% | 10.30% | 2.10% | 10.60% | 5.70% | 3.40% | 1.40% |
| 2023년 | 인원 | 1,238,495 | 1,097,913 | 52,913 | 98,015 | 297,796 | 499,584 | 123,971 | 25,634 | 140,582 | 75,552 | 47,410 | 17,620 |
|  | 비율 | 100% | 88.60% | 4.30% | 7.90% | 24.00% | 40.30% | 10.00% | 2.10% | 11.40% | 6.10% | 3.80% | 1.40% |
| 2022년 | 인원 | 1,160,850 | 1,019,130 | 49,946 | 94,233 | 278,520 | 459,316 | 113,842 | 23,273 | 141,720 | 74,878 | 50,385 | 16,457 |
|  | 비율 | 100% | 87.80% | 4.30% | 8.10% | 24.00% | 39.60% | 9.80% | 2.00% | 12.20% | 6.50% | 4.30% | 1.40% |
| 2021년 | 인원 | 1,097,462 | 953,511 | 47,800 | 92,461 | 261,047 | 423,595 | 106,107 | 22,501 | 143,951 | 74,838 | 53,700 | 15,413 |
|  | 비율 | 100% | 86.90% | 4.40% | 8.40% | 23.80% | 38.60% | 9.70% | 2.10% | 13.10% | 6.80% | 4.90% | 1.40% |
| 2020년 | 인원 | 1,007,423 | 857,984 | 43,040 | 86,998 | 238,697 | 378,126 | 91,960 | 19,163 | 149,439 | 76,481 | 58,659 | 14,299 |
|  | 비율 | 100% | 85.20% | 4.30% | 8.60% | 23.70% | 37.50% | 9.10% | 1.90% | 14.80% | 7.60% | 5.80% | 1.40% |
| 출처: 국민건강보험공단 홈페이지. 장기요양보험 경영실적, 장기요양 등급 판정 현황. 2024.9.30. | | | | | | | | | | | | | |

- 국민건강보험공단의 2023 노인장기요양보험 통계연보에 따르면 이러한 장기요양보험 수급자는 2023년 12월 말을 기준으로 총 인정자는 1,097,913명이고 이는 65세 이상의 노인인구 대비 11.1%에 해당하며 이러한 수치는 5년간 매년 증가하는 추세를 보이고 있음.

○ 연령이 증가하면서 생리적 노화와 질병 누적에 따른 변화가 함께 나타남에 따라 노인병은 애매하고 복잡한 질병 양상을 보이는데, 이러한 특성을 노인증후군(Geriatiric Syndrome)이라 지칭함.

- 기능 저하 노인의 경우 상대적으로 노인증후군의 위험이 높아지며, 노인증후군의 악순환이 시작되면 이전 수준으로 기능을 회복시키는 것은 매우 어려움.

- 2013년 아시아태평양 지역의 노인병 전문 학자들의 견해를 중심으로 연구한 바에 따르면, 노인증후군에는 노쇠, 근감소증, 거동장애, 보행장애, 욕창, 치매 등이 포함됨.

다. 한의계의 통합돌봄과 재택의료센터 참여 현황

○ 2019년 지역사회 통합돌봄사업이 시작된 이후 한의사는 기능저하 노인의 건강을 관리하기 위해 통합돌봄과 재택의료센터 사업에 활발하게 참여하고 많은 성과를 만들어왔음.

- 한의 돌봄 사업은 지역사회 통합돌봄사업에서 2021년 13개 지역, 2022년 20개 지역, 의료·돌봄 통합지원 시범사업으로 전환된 2023년 25개 지역 등 지속적으로 확대되어왔으며, 매년 우수 사례를 발굴하고 성과를 공유하는 등 정기적인 모니터링 또한 이루어지고 있음.

- 한의 돌봄에 대한 수요에 따라 장기요양 재택의료센터 시범사업에서도 1차에는 28개 의료기관 중 3개 한의원이 참여한데 비해 2차에서는 95개 의료기관 중 24개 한의원이 선정됨.

| 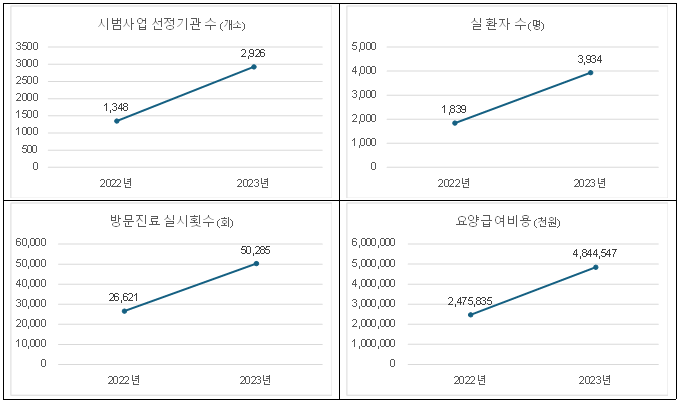  **<일차의료 한의방문진료 수가 시범사업 ‘22-’23>** | 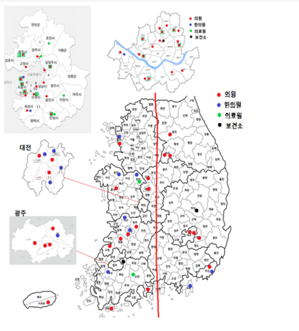  **<한의 장기요양 재택의료센터 현황>** |
| --- | --- |

○ 장기요양보험 대상자는 한의 장기요양 재택의료센터(이하 한의 재택의료센터) 등을 통하여 한의 돌봄 서비스를 제공 받을 수 있음.

- 본 연구진은 선행 연구인 「한의 장기요양 재택의료센터 안내서 개발 연구」(한국한의약진흥원, 2024)의 일환으로 한의 재택의료센터 방문진료를 수행하는 한의사를 대상으로 수행 된 설문조사를 통하여 한의를 이용하는 기능저하 노인의 진료 현황과 질환 특성에 대해 파악함.

- 22개 한의 재택의료센터에 등록된 765명의 장기요양보험 수급자를 대상으로 진행된 설문조사에서 대상자들은 주로 만성병/통증관리, 신체기능 재활, 노인병 증후군에 대한 문제를 보유한 경우 한의사의 방문진료를 요청하였음.

- 방문진료시 한의사가 현장에서 실제 다루게 되는 증상 및 관리를 복수응답으로 조사한 결과 ‘근골격계 통증 완화’가 32.9%으로 가장 많은 것으로 나타났으며 ‘고혈압, 당뇨, 이상지질혈증 등 대사성 질환 모니터링 및 관리’ 13.2%, ‘마비, 구축 관리 등 신체기능 재활’ 11.7% 순 것으로 확인되었음.

- 또한 ‘요실금 및 배뇨 관리’ 6.7%, ‘치매, 수면장애, 우울, 불안 등 정신장애 완화’ 6.2%, ‘욕창 등 피부질환 관리’ 3.6% 등 대상자의 다수 질환과 증상에 대한 진료 및 관리를 수행하는 것으로 확인됨.

**2) 연구의 필요성**

가. 연구의 필요성

○ 정부는 2026년 노인 의료·돌봄 통합지원 사업의 본사업 시행을 계획하고 있으나 한의계에는 연구가 부족한 실정임.

- 한의계의 적극적인 참여에도 불구하고 한의 돌봄에 대한 성과를 체계적으로 평가한 연구가 부재하며, 현장에서의 높은 참여도에 비해 한의의 역할을 직접적으로 확인할 수 있는 정책적 근거 자료가 부족함.

- 이에, 한의 돌봄의 사업 성과를 종합하여 제시함으로써 한의 참여의 근거를 마련할 필요가 있음.

○ 정책적 근거 마련을 위하여 한의원 기반 재택의료센터 시범사업의 체계적 평가가 필요함.

- 본 사업 시행에 앞서 한의학적 접근의 특성과 효과를 다각도로 평가하여 표준화된 한의 재택의료 모델 개발 및 확산의 토대를 마련해야 함.

- 일상생활 수행능력, 환자 건강 지표, 만족도를 함께 평가함으로써 서비스의 전반적 효과를 정량적으로 평가할 필요가 있음.

**2. 연구 목적**

**1) 연구 목적**

○ 1차 목적

- 한의과 재택의료센터에서 치료받은 기능 저하 노인의 일상생활 수행능력 지표의 변화를 파악함.

○ 2차 목적

- 한의과 재택의료센터에서 치료받은 기능 저하 노인의 노쇠 및 만성 통증 지표의 변화를 파악함.

- 치료 결과에 영향을 미치는 예측요인(환자 특성, 의료기관 특성 등)을 분석함.

- 등록된 재택의료센터의 의료서비스 현황 및 이용 환자의 인구학적·임상적 특성을 기술 통계적으로 파악함.

**3. 연구책임자, 공동연구자, 담당자의 성명과 직명**

**1) 연구책임자**

| **성명** | **소속** | **직위** | **연락처** | **역할** |
| --- | --- | --- | --- | --- |
| 김범석 | 중동한의원 | 원장 | 010-2772-1075/ds2jqu@hanmail.net | 연구총괄,  연구대상자 모집, 동의 획득, 자료수집 |
| 김권희 | 건강한마을한의원 | 원장 | 010-9932-1015/pine0426@daum.net | 연구대상자 모집,  동의 획득,  자료수집 |
| 방호열 | 동방신통부부한의원 | 원장 | 010-3554-9750/rhrnal@daum.net | 연구대상자 모집,  동의 획득,  자료수집 |
| 김창훈 | 해맑은한의원 | 원장 | 010-4727-2533/lovehadela@naver.com | 연구대상자 모집,  동의 획득,  자료수집 |
| 김정철 | 김정철한의원 | 원장 | 010-8954-7958/eegol@hanmail.net | 연구대상자 모집,  동의 획득,  자료수집 |
| 심희준 | 서화한의원 | 원장 | 010-7479-3393/tow10002@gmail.com | 연구대상자 모집,  동의 획득,  자료수집 |

**2) 공동연구자**

| **성명** | **소속** | **직위** | **연락처** | **역할** |
| --- | --- | --- | --- | --- |
| 김동수 | 동신대학교 한의과대학 | 조교수 | 010-6736-5661/dskim20@dsu.ac.kr | 연구계획, 연구주관, 연구자문 |
| 진한빛 | 동신대학교 한의과대학 | 대학원생 | 010-9686-8655/hanbitjin22@gmail.com | 연구계획, 연구실무 담당자 |
| 전형선 | 원광대학교  한의과대학 | 대학원생 | 010-5548-1423/hs14231423@naver.com | 연구계획,  연구실무 담당자 |
| 송수민 | 청년한의사회 | 연대사업국장 | 010-4408-7719/s01044077719@gmail.com | 연구실무 담당자 |
| 배예린 | 동신대학교 한의과대학 | 연구원 | 010-4545-1240/dpfls1240@gmail.com | 연구실무 담당자 |
| 안은지 | 동신대학교 한의과대학 | 대학원생 | 010-9104-1515/ahneunji1015@daum.net | 연구실무 담당자 |

**4. 연구실시 기관명 및 주소**

**1) 연구 자료 수집**

| **의료기관 명** | **주소** |
| --- | --- |
| 중동한의원 | 경기도 부천시 원미구 석천로 181 원흥빌딩 2층 |
| 건강한마을한의원 | 전북 전주시 완산구 장승배기로 168 3층 |
| 동방신통부부한의원 | 경남 거제시 고현로 111 1층 |
| 해맑은한의원 | 충청남도 천안시 서북구 불당21로 65 3층 |
| 김정철한의원 | 대전광역시 중구 천근로 12-1 |
| 서화한의원 | 경기 고양시 덕양구 충장로 126 행신스퀘어 4층 |

| ※ 재택의료센터 기관 선정 이유  1. 재택의료센터란?  ‧ 장기요양 재택의료센터 시범사업은 장기요양 1~4등급 수급자 중 거동이 불편하여 재택의료가 필요하다고 한의사가 판단한 자를 대상으로 하며, 한의사 간호사 사회복지사 등이 주기적으로 가정을 방문하여 방문진료, 간호 및 지역사회 돌봄 연계 등을 제공함.  ‧ 한의사는 월 1회 이상 방문진료, 간호사는 월 2회 이상 방문간호를 제공하며 사회복지사는 주기적 상담을 통한 요양․돌봄 수요 발굴 및 서비스연계함. 한의사의 방문진료 서비스 제공 내용은 다음과 같음.  ① (진찰) 문진(問診), 문진(聞診), 망진(望診), 촉진(觸診), 청진(聽診), 타진(打診), 안진(按診), 맥진(脈診) 등  ② (처방) 「한약제제 급여목록 및 상한금액표」상의 한약제제* * 예: 단미엑스제제(갈근엑스산, 감초엑스산, 당귀엑스산 등), 단미엑스혼합제(가미소요산, 갈근탕, 구미강활탕 등)  ③ (질환관리) 주증 및 동반질환에 대한 관리(침술, 구술, 부항술 등)  ④ (검사) 인성검사 등 한방 검사  ⑤ (의뢰) 필요 시 적절한 전문 의료기관으로의 의뢰  ⑥ (교육상담) 환자 상태 설명 및 질환 정보 제공, 건강관리 등에 대한 환자・보호자교육 등  2. 참여 기관 선정 이유  ‧ 본 연구에 참여하는 6개 한의과 재택의료센터는 모두 ‘한의재택의료학회*’ 소속으로, 한의 돌봄 및 건강돌봄 기반 연구에 대한 이해도와 실천 경험이 높은 기관들임. 이들은 과거 본 연구팀이 수행한 한의 돌봄 관련 연구에서 핵심적인 협력 파트너로 참여한 경험이 있으며, 연구 수행 역량과 참여 의지가 검증된 기관으로서 선정됨.  *한의재택의료학회는 한의약 기반의 건강돌봄 진료모델을 구축하고자 하는 실무 임상의 및 연구자로 구성된 Practice-Based Research Network(PBRN)로, 2025년 7월 기준 총 109명의 회원으로 구성되어 있으며, 정기적인 스터디와 외부 특강, 실무 공유 세션을 통해 연구 기반을 확대하고 있는 단체임. |
| --- |

**2) 연구 자료 분석**

○ 동신대학교 예방의학교실 (전남 나주시 동신대길 120-9 동신대학교 대정4관 303호)

**5. 연구 지원기관**

○ 한국보건산업진흥원 (충북 청주시 흥덕구 오송읍 오송생명2로 187 보건의료행정타운)

**6. 연구 기간**

○ IRB 승인일~2028.12.31

**7. 연구대상자**

○ 본 연구의 환자모집기관 6개소 재택의료센터 환자 중 선정/제외 기준을 만족하는 자

- 장기요양 1등급은 '심신의 기능 상태 장애로 일상생활에서 전적으로 다른 사람의 도움이 필요한 자‘, 2등급은 '심신의 기능 상태 장애로 일상생활에서 상당 부분 다른 사람의 도움이 필요한 자'로 등급 판정 자체가 일상생활능력이 현저히 저하되어 있음을 의미함

- 따라서, 상태 변화가 미미할 것으로 예상되어 장기요양 3, 4등급인 자를 연구 대상으로 함

| **선정기준** | - 재택의료센터에서 치료를 받은 자  - 65세 이상 노인  - 장기요양 3, 4등급인 자  - 연구에 대해 명확한 설명을 듣고 충분히 이해한 후, 자의 또는 법정대리인의 의사로 연구 참여에 결정하고 동의서에 서명한 자 |
| --- | --- |
| **제외기준** | - 1년 동안 추적관찰이 불가능할 것으로 예상되는 자 (예: 3개월 이내의 기대여명을 가진 말기 질환자, 타 지역 이사 예정자 등)  - 연구 참여에 영향을 줄 수 있는 다른 임상연구에 참여 중인 자  - 급성기 질환으로 상태가 불안정하여 재택의료보다 입원치료가 우선적으로 필요하다고 판단되는 자  - 심각한 인지기능 저하 또는 의사소통 장애로 인해 본인 및 법정대리인을 통해서도 연구 평가 수행이 어렵다고 판단되는 자 |

**8. 예상 연구대상자 수와 산출 근거**

**1) 목표 등록 인원**

○ 첫 환자등록 시점부터 1년 동안 6개소의 연구대상자 모집 기관에서 경쟁적 방식으로 250명을 모집하며, 1년 동안 추적관찰 후 최종적으로 약 200 person-year을 목표로 함.

**2) 표본 추출 방법**

○ 레지스트리 연구는 미리 정해진 통계적 가설을 검정할 필요가 없으므로, 사전 표본 크기 계산이 필요하지 않으나, 연구 상황을 고려하여 현실적으로 가능한 범위 내에서 표본 수를 산정함.

○ 현실적 모집 가능성을 계산하기 위해 2024년 9월 수치를 참고하여 대상자 수를 산출함.

- 레지스트리 연구대상자 모집 기관 6곳은 전국적으로 분포되어 있으며, 2024년 9월을 기준으로 참여기관들의 장기요양 3, 4등급 환자 수를 합산하면 370명 가량임. 본 연구의 환자 모집 기간인 2025년-2026년에도 동일한 환자 수가 유지된다는 것을 가정하여 대상자 수를 산출함.

- 370명의 70%인 259명이 연구에 참여한다고 가정하고 20%의 탈락률을 고려했을 때, 약 200 person-year을 목표로 함. 이때 탈락률 20%는 연구 기간 중 발생할 수 있는 대상자의 합병증, 상태 악화로 인한 입원 및 시설 입소, 사망 등 지속적인 연구 참여가 어려운 경우를 모두 포함하여 산정한 것임.

- 재택의료센터는 방문 진료를 통해 환자에게 서비스를 제공하므로, 참여율은 높고 중도 탈락자는 적을 것으로 예상됨.

| **참여기관** | **건강한마을 한의원** | **중동 한의원** | **동방신통부부 한의원** | **해맑은 한의원** | **김정철 한의원** | **서화한의원*** | **총 인원** |
| --- | --- | --- | --- | --- | --- | --- | --- |
| 2024년 9월  환자 수 | 98 | 78 | 73 | 61 | 60 | - | 370 |
| 등록 목표  (총 환자의70%) | 69 | 55 | 51 | 42 | 42 | - | 259 |
| 최종 추적 목표  (탈락률 20% 추정) | 55 | 44 | 40 | 33 | 33 | - | 205 |

* 2025년 신규선정된 기관으로, 대상자 수 산정 불가함

○ 추가로 본 연구에서는 치료에 영향을 미치는 요인에 대한 통계분석을 수행하여 치료 반응자를 확인하고자 하며, 이를 토대로 연구대상자 수를 산출함.

- 연속형 변수인 K-ADL에 대한 다변량 로지스틱 회귀분석을 수행하고자 하며, 치료 성공에 영향을 미치는 공변량은 10개로 사전에 선정함.

- 공변량은 재택의료센터 포괄평가 기록지의 항목 중 성별, 연령, 심혈관질환 여부, 당뇨 여부, 고혈압 여부, 고지질혈증 여부, 치료 전 K-FRAIL 점수, 치료 전 Five-times chair and stand test (5CST) 점수, 치료 전 Timed up and go test (TUG test) 점수, 치료 전 10계단 올라가기 능력 점수임.

- 하나의 변수에 일반적으로 20명의 대상자가 필요하므로, 이에 따라 10*20=200명의 연구대상자를 필요로 함.

**9. 연구대상자 모집**

○ 연구대상자 모집 방법

- 본 연구는 환자등록연구로, 연구대상자 모집 과정은 일반적인 진료 과정의 일환임.

- 재택의료센터 진료는 방문진료를 통해 이루어지기 때문에, 연구참여기관의 원장이 공동연구자로서 대상자의 집에 방문하여 IRB 승인을 받은 연구 설명문과 동의서를 대상자 혹은 법정대리인에게 충분히 설명한 후 모집함.

- 또한, 본 연구에 대한 모집 광고문을 연구 대상자 모집 기관의 원내 게시판에 부착하며 홈페이지상에 팝업창을 통해 게시함.

- IRB 승인일 이후 1년간 모집하며, 모든 모집 과정은 김동수 공동연구자가 주관하여 진행함.

**10. 연구대상자 동의**

**1) 동의서**

○ 공용기관생명윤리위원회의 승인을 받은 2종의 설명문 및 동의서를 사용할 예정임.

- 연구 참여 동의서: 본 환자등록연구 참여 자체에 대한 동의를 구하는 서식

- 개인정보 제3자 제공 및 2차 연구 이용 동의서: 연구를 통해 수집된 연구대상자의 정보를 한의약진흥원에 제공하고, 향후 다른 연구에 2차적으로 활용하는 것에 대한 동의를 별도로 구하는 서식

○ 연구자는 연구대상자가 '연구 참여 동의서'에만 동의하고 '개인정보 제3자 제공 및 2차 연구 이용 동의서'에는 동의하지 않더라도 본 환자등록연구에 참여할 수 있음을 명확히 설명해야 함.

- 이 경우, 해당 연구대상자의 정보는 본 연구에만 사용되며 한의약진흥원에는 제공되지 않음.

○ 연구대상자의 동의는 연구 참여기관 소속 연구진이 대상자에게 직접 설명하고 서면 동의를 받는 방식으로 이루어질 예정임.

- 동의 획득 장소는 대상자의 자택 내에서 안정적이고 조용한 환경을 확보하여, 연구진과 대상자 간 충분하고 원활한 의사소통이 가능한 공간에서 시행할 예정임.

- 이 과정에서 참여자의 이름 등 개인 식별정보는 연구진 외 접근이 불가하도록 안전하게 관리해야 함.

- 연구진은 다음과 같은 내용을 연구대상자에게 충분히 설명해야 함.

• 연구의 목적 및 배경

• 연구 절차 및 예상 소요 시간

• 수행되는 검사 및 자료 수집 내용

• 개인정보 보호 및 비밀 유지에 대한 조치

• 수집된 데이터가 공동연구기관으로 이양되어 분석될 수 있음

• 연구 참여는 자발적인 결정이며, 언제든지 불이익 없이 철회 가능함

- 대상자의 질의가 있을 경우, 연구진은 충분한 시간을 두고 설명하고 성실히 응답해야 함.

- 연구대상자가 설명을 충분히 듣고 자발적으로 참여 의사를 표명한 경우, 연구진은 해당 당일에 서면 동의서를 작성·서명받은 후 연구에 참여시키며, 강제나 유도 없이 자율적으로 결정하도록 해야 함.

- 본 서면 동의는 전체 연구기간 동안의 참여에 대한 동의를 의미하나, 연구기간 중 대상자의 요청이나 동의능력의 변화 여부에 따라 추가로 재동의서를 받을 수 있으며, 이에 따라 필요한 경우에는 재동의 절차를 진행해야 함.

**2) 연구대상자의 동의 능력 평가**

○ 연구대상자의 동의 능력 평가에 대한 계획

- 1) 연구대상자가 연구와 관련된 정보를 이해하는지 여부, 2) 연구에 대한 정보를 논리적으로 다룰 수 있는지 여부, 3) 연구에 참여하고 싶어하는지 아닌지에 관한 자신의 선택을 명확하게 의사표현하는 능력이 있는지 여부 등을 종합적으로 판단함.

- 위 동의 능력 평가에 근거하여 충분한 동의 능력이 있다고 판단되는 경우 연구대상자 본인의 동의를 받음.

- 위 평가에 근거하여 연구참여에 대한 동의가 어렵다고 판단되면, 취약한 대상자로 분류하여 연구대상자 본인의 서면 승낙 및 법정대리인의 동의를 받음.

- 법정대리인의 동의 능력 평가가 필요한 경우는 연구 참여기관 연구 한의사의 진료 등을 통하여 평가를 진행함.

- 법정대리인의 동의 능력이 저하되어 있으면 다른 법정대리인의 동의를 받거나, 동의 능력이 있는 법정대리인이 없는 경우 해당 환자는 등록하지 않음.

- 법정대리인 동의는 법정대리인이 작성해야하며, 이 경우 법정대리인의 동의를 증명하기 위한 가족관계증명서 등의 문서는 취약한 연구대상자의 권리 보호를 위해 적법한 대리인으로부터 동의를 얻었음을 객관적으로 입증하기 위한 필수 자료이므로 수집 및 보관함.

• 가족관계증명서 등의 제 3자의 개인정보는 법정 대리인 확인 후 즉시 문서 파쇄기를 이용하여 완전히 폐기함.

○ 연구대상자에게 대한 위험을 최소화하기 위한 절차

- 연구참여기관의 원장이 매 방문진료 시 대상자의 인지 기능이나 의사소통 능력에 현저한 변화가 있다고 판단할 경우 수시로 동의 능력을 재평가함.

• 최초 본인 동의로 연구에 참여했으나, 추적관찰 중 동의 능력이 저하되어 법정대리인의 동의가 필요하게 된 경우, 대상자 혹은 법정대리인이 동의 내용에 대한 재설명을 요청하는 경우 재동의 취득 사유가 됨

- 이러한 경우 최초 동의 획득 절차와 동일한 방식으로 법정 대리인의 동의를 받음.

- 법정 대리인의 동의 능력이 저하된 경우, 동의 능력이 있는 다른 법정대리인의 동의를 받도록 하며, 동의 능력이 있는 법정대리인이 없는 경우 해당 환자는 중도 탈락시킴.

- 언제든지 연구의 지속과 중단을 결정할 수 있다는 사항, 연구 참여 중단으로 인한 불이익이 없다는 사항을 반복적으로 알림.

**11. 연구 방법**

**1) 연구 디자인**

○ 본 연구는 레지스트리 기반 관찰연구로, 특정 인자에 노출된 환자를 대상으로 균일한 형식의 데이터를 지속적이고 체계적으로 수집하여, 장기적인 건강 결과 및 서비스 제공 효과를 평가하고자 함.

- 레지스트리 연구는 일반적으로 특정 질환의 특성, 치료의 임상적 효과, 서비스의 질 등을 파악하고 개선하기 위해 수행되며, randomized controlled trial과 같이 정형화된 틀 없이 연구진의 목적에 따라 변형 가능하다는 장점이 있음.

- 질환 기반 레지스트리(disease registry)와 보건의료 서비스 기반 레지스트리(health services registry) 등 다양한 형태가 존재하며, 단일 목적이 아닌 여러 목적을 결합하여 운영될 수도 있음.

- 본 연구는 한의과 재택의료센터의 서비스를 받은 환자 등록을 목적으로 하며, 등록된 환자 중 노쇠와 만성통증에 대한 평가가 이루어질 예정이므로 health services registry와 disease registry의 목적을 결합한 레지스트리로 운영함.

○ 본 연구에서는 포괄평가 기록 시점을 기준으로 한의 재택의료센터 서비스 노출 여부를 정의하고, 아래와 같이 혼합형(후향 + 전향) 자료 수집 구조를 적용함.

- 재택의료센터는 환자 첫 진료시 장기요양 재택의료센터 시범사업 지침의 제5호서식인 ‘장기요양 재택의료센터 포괄평가 및 케어플랜 기록지’를 활용하여 초기 상태를 평가함.

- 따라서, 모든 환자는 첫 포괄평가 기록이 남아있으며, 그 시점을 노출 시점으로 정의함.

- 신규 재택의료센터 환자의 경우, 포괄평가 기록지를 포함한 모든 측정 변수를 전향적으로 수집함.

- 기존 재택의료센터 환자의 경우, 첫 포괄평가 시기를 재택의료센터 치료에 대한 노출로 판단하여 과거 포괄평가 기록지를 후향적으로 수집하며, 연구를 위한 측정 변수 및 연구 기간 중의 포괄평가 기록지는 전향적으로 수집함.

**2) 기능저하, 노쇠, 만성통증 정의**

○ 기능저하의 정의

- 기능이란 개인의 신체기능, 활동수행, 사회참여 능력을 의미하며, 기능저하는 이 능력이 손상되거나 제한된 상태로 정의됨.

- 장기요양인정조사표에 따르면 신체기능(12문항), 인지기능(7문항), 행동변화(14문항), 간호처치(9문항), 재활(10문항) 영역에서 신청인의 일상생활 수행 능력과 기능저하 정도를 종합적으로 평가하여 장기요양 등급판정에 활용함.

- 이러한 등급판정은 신체적·인지적 기능 저하를 반영함으로 장기요양등급자는 기능저하 노인으로 정의할 수 있음.

- 본 연구에서는 연구대상자의 기능저하 수준 변화를 K-ADL(7점: 완전 독립, 21점: 완전 의존)로 측정할 예정임.

○ 노쇠

- 노쇠란 노화로 인한 전반적인 기능저하로 인해 신체의 회복력과 생리적인 예비능력이 감소하여 외부자극에 적절히 대응하지 못하는 취약상태로, 질병, 기능장애, 의존, 사망 위험이 증가하는 상태를 말함.

- 본 연구에서는 연구대상자의 노쇠 수준 변화를 K-FAIL로 평가할 예정이며, 0점인 경우 정상, 1-2점인 경우 전노쇠, 3-5점인 경우 노쇠로 평가함.

○ 만성통증

- WHO는 ICD-11에서 만성통증을 “3개월 이상 지속되거나 반복적으로 나타나는 통증으로 정의하며, 명확한 원인이 있든 없든 간에 삶의 질을 저하시킬 수 있는 질환 상태”로 정의함.

- 만성통증은 일정 기간 이상 지속되거나 손상이 회복된 이후에도 사라지지 않는 통증으로, 증상이 아닌 독립적인 질환으로 여겨짐.

- 만성통증은 지속적인 진통제 사용을 높이며, 만성통증 환자의 약 75%가 최소한 하나의 진통제를 처방받았음을 보고함.

- 본 연구에서는 만성통증을 ICD-11의 정의에 따르며, Numerical Rating Scale(NRS)로 평가할 예정임.

**3) 연구대상자 등록**

○ 동의를 받는 기관과 순서에 따라 식별코드를 부여하여 기록하며, 대상자의 이름은 앞 3자리까지 이니셜로 표기함.

- 실시기관 코드-실시년도-등록된 순서로 표기함.

- 표기 예시는 다음과 같음; JDC-2026-015: 중동한의원 2026년도 15번째 연구 참여 환자

| **의료기관 명** | **코드** |
| --- | --- |
| 중동 한의원 | JDC |
| 건강한마을 한의원 | GGH |
| 동방신통부부 한의원 | DBS |
| 해맑은 한의원 | HME |
| 김정철 한의원 | KJC |
| 서화 한의원 | SHC |

| **대상자 식별코드** | **대상자 이니셜** |
| --- | --- |
| \|  \|  \|  \| \| --- \| --- \| --- \|   **– 202_ -**   \|  \|  \|  \| \| --- \| --- \| --- \| | \|  \|  \|  \| \| --- \| --- \| --- \| |
| □ NA (Screening Fail) |  |

**4) 연구 프로세스**

| **스크리닝** | 연구 참여 동의 | - 연구대상자 및 법정대리인 동의서 취득 |
| --- | --- | --- |
|  | 스크리닝 조사 | - 선정/제외기준 확인  - 대상자 번호 부여  - 개인정보(성명, 생년월일, 성별, 주소) |
| **측정1** | 기저조사 | - K-Activities of Daily Living(K-ADL, 일상생활수행능력)  - K-FRAIL (노쇠)  - Numerical Rating Scale(NRS, 통증)  - 약물 투여력 |
| **측정2** | 1년 후  추적조사 | - K-Activities of Daily Living(K-ADL, 일상생활수행능력)  - K-FRAIL (노쇠)  - Numerical Rating Scale(NRS, 통증)  - 약물 투여력  - Client Satisfaction Questionnaire(CSQ, 만족도) |
| **지속측정** | 1년 간  추적조사 | - 이상반응  - 장기요양 재택의료센터 방문점검 및 업무 기록지(의사, 간호사, 사회복지사)  - Numerical Rating Scale(NRS, 통증) |
| **포괄평가** | 정기조사 | - 장기요양 재택의료센터 포괄평가 및 케어플랜 기록지 |

| **측정** | **Before**  **the study** | **Screening*** | **Measure 1** | **Measure 2** |
| --- | --- | --- | --- | --- |
| **Months** |  | **0** | **0**  **(+30일)** | **12**  **(+-30일)** |
| **동의서 취득** |  | ● |  |  |
| **선정/제외기준 확인** |  | ● |  |  |
| **대상자 번호 부여** |  | ● |  |  |
| **개인정보**  **(성명, 생년월일, 성별, 주소)** |  | ● |  |  |
| **포괄평가 기록지**** |  | | | |
| **K-ADL K-FRAIL, NRS, 약물 투여력** |  |  | ● | ● |
| **Client Satisfaction Questionnaire** |  |  |  | ● |
| **이상반응, 방문점검 및 업무 기록지 (의사, 간호사, 사회복지사),**  **NRS** |  |  |  | |
| * 스크리닝에서 선정기준에 부합할 경우 1차 측정을 수행할 수 있음.  ** 연구 대상자마다 1년에 1회 정기적으로 측정하므로, 의료 기관의 측정 시기에 맞추어 수집함. 재택의료센터의 기존 환자는 후향적 데이터까지 수집하며, 신규 환자는 전향적 데이터만 수집함. | | | | |

○ 연구대상자의 일정은 다음과 같음.

- 연구 참여 의지를 밝히면 대상자 동의서 취득, 선정/제외기준 확인, 대상자 번호 부여 및 개인정보(성명, 생년월일, 성별, 주소)를 확인함.

- 환자 상태 평가를 위한 기저조사(K-ADL, K-FRAIL, NRS, 약물 투여력) 실시함.

- 1년간 재택의료센터의 서비스를 받으며, 매 방문시 방문점검 기록지(의사, 간호사, 사회복지사), NRS, 이상반응을 기록함.

• 본 연구는 관찰연구로, 연구 참여로 인한 추가적인 의료 방문은 없음. 연구 기간 동안의 방문 주기는 각 대상자의 상태에 따라 담당 의료진의 판단하에 수립된 재택의료센터의 케어플랜을 따르게 됨.

• 그러나 최소한의 방문 기준으로 장기요양 재택의료센터 시범사업 지침에 따라 한의사는 월 1회, 간호사는 월 2회 이상 방문하게 됨.

- 치료 1년 후 환자 상태 평가를 위한 추적조사(K-ADL, K-FRAIL, NRS, 약물 투여력)와 만족도 평가 진행함.

• 일상생활 수행능력(K-ADL)이나 노쇠(K-FRAIL)는 그 특성상 점진적으로 변화하므로, 임상적으로 의미 있는 변화를 관찰하기 위한 최소 기간인 1년의 추적 기간을 설정함.

- 포괄평가 기록지는 기저조사와 추적조사의 일정과 달리, 연구 참여기관의 프로세스에 따라 측정함.

- 연구 종료 후에도 재택의료센터의 치료는 지속됨.

○ 연구에 소요되는 시간은 다음과 같음.

- 연구기간 동안 기저조사와 추적조사, 최소 1회 이상의 방문진료를 받아야 하며, 방문진료는 약 30분 소요됨.

- 기저조사와 추적조사는 약 1시간이 소요됨.

○ 연구의 종료는 다음과 같음.

- 연구대상자가 등록 후 1년 시점의 최종 추척조사를 마친 경우 연구 참여는 종료됨.

**12. 관찰 항목**

**1) 관찰 항목**

○ 개인정보(성명, 생년월일, 성별, 주소)

- 본 연구는 2차 자료 결합을 위해 성명, 생년월일, 성별, 주소를 결합키로 수집해야 하며, 해당 정보는 진료 과정에서 필수적으로 수집되는 항목으로, 연구 참여 여부와 무관하게 확보 가능함.

- 수집된 개인정보는 연구책임자 및 공동연구자만 열람 가능하며, 자료는 시건장치가 설치된 캐비닛, 암호로 보호되는 컴퓨터 등에 보관하고 허가받은 사람만 접근 가능하도록 조치함.

○ 포괄평가지, 방문점검 및 업무 기록지(의사, 간호사, 사회복지사) (별첨1-4)

- 장기요양 재택의료센터 시범사업 지침의 제5호서식인 ‘장기요양 재택의료센터 포괄평가 및 케어플랜 기록지’와 제6호, 제7호, 제8호서식인 ‘장기요양 재택의료센터 방문점검 기록지(의사)’, 장기요양 재택의료센터 방문점검 기록지(간호사)’, ‘장기요양 재택의료센터 업무 기록지(사회복지사)’를 사용함.

- 포괄평가 기록지는 1년에 1회 작성하며 방문점검 및 업무 기록지는 매 방문진료 시 기록함.

- 포괄평가지 내 장기요양인정번호와 유효기간, 보호자 정보는 수집하지 않으며, 방문점검 및 업무 기록지의 장기요양인정번호 또한 삭제하여 수집하지 않음.

- 포괄평가지, 방문점검 및 업무 기록지의 활용 계획은 다음과 같음.

• 대상자 특성 분석: 포괄평가 기록지를 활용하여 연구 시작 시점의 인구학적 정보, 동반 질환, 신체 및 인지 기능, 복용 약물 등 연구대상자의 다각적인 기저 특성을 파악하여 향후 재택의료 서비스 효과 분석의 기반 자료로 활용함.

• 의료 서비스 내용 파악: 방문점검 및 업무 기록지를 통해 연구 기간 동안 제공된 진료의 종류, 빈도, 소요 시간, 처치 내용 등 구체적인 서비스 내용을 정량적으로 분석함.

| ※ 수집하는 포괄평가지 및 방문점검 기록지 정보  ‧ 포괄평가지: 방문일, 기관정보, 대상자 정보(성명, 생년월일, 성별, 장기요양등급, 주소), 건강/질병 상태(해결 요청 주요 문제, 치료중인 동반 질환, 투약 내용 등), 신체상태(보행능력 및 근력 검사, 영양상태 및 식사행위 등), 정신상태(인지기능, 우울 등), 자립생활 가능성(의식수준, 신체기능, 인지기능 등), 의료처치 및 건강관리 필요 항목, 사회/환경 평가(일상생활 도움 필요 여부, 경제상황, 사회관계 등), 서비스 이용현황(의료서비스, 장기요양서비스 등)  ‧ 방문점검 기록지(의사): 기관정보, 대상자 정보(성명, 생년월일, 성별, 장기요양등급, 주소), 서비스 제공시간, 방문사유, 신체기능 자립성, 인지기능 자립성, 진찰 및 상담 내용, 침습적 처치 내용, 간호지시, 향후계획  ‧ 방문점검 기록지(간호사): 기관정보, 대상자 정보(성명, 생년월일, 성별, 장기요양등급, 주소), 방문 사유, 서비스 제공시간, 동행자, 방문내용(투약관리 포함), 건강상태, 혈압/맥박, 체온/혈당, 체중 변화, 섬망, 낙상, 소변/대변실금, 향후계획  ‧ 방문점검 기록지(사회복지사): 기관정보, 대상자 정보(성명, 생년월일, 성별, 장기요양등급, 주소), 상담 내용, 지역사회연계 |
| --- |

○ K-ADL (별첨5)

- 2002년 보건복지부 발주로 대한노인병학회 노인기능평가연구회에서 우리나라의 문화에 맞도록 수정하여 개발한 한국형 일상생활 측정도구임.

- 7개의 질문으로 이루어져 있으며 점수가 높을수록 기본적인 일상생활을 스스로 잘 하는 것을 말하고, 0점에 가까울수록 타인의 도움이 더 많이 필요함을 나타냄.

○ K-FRAIL (별첨6)

- 노쇠의 진단에 많이 사용되며, 일차의료 환경에서 적용하기에 적합한 도구임.

- 다섯 가지 노쇠 기준으로 이루어져 있으며 합산 점수에 따라 세 가지 노쇠 단계로 분류함; 노쇠하지 않음(점수 0), 노쇠 전 단계(점수 1-2), 노쇠(점수 3-5).

○ NRS (별첨7)

- Visual Analogue Scale를 수치화하여 0-10까지 숫자로 표현한 것으로, 0은 ‘통증이 전혀 없는 상태’이고 10은 ‘상상할 수 있는 가장 심한 통증’으로 가정하도록 하여 현재 통증의 정도를 숫자로 말하도록 하는 지표임.

- 만성통증 환자들에서 NRS가 이해하기 쉽고 수행하기 쉬워 VAS보다 더 선호한다는 연구 결과가 있음.

○ CSQ (별첨8)

- 재택의료에 대한 환자 만족도 연구에서 사용한 설문지를 사용함.

- CSQ는 선행연구를 통해 높은 내적 일치도를 갖고 있으며, 환자의 증상감소 및 지속치료율과 높은 상관관계가 있음이 알려졌음.

**2) 측정 방법**

○ 설문지

- K-FRAIL, NRS, K-ADL, 포괄평가 기록지, 방문점검기록지(의사, 간호사, 사회복지사)는 사전에 교육받은 연구진이 시행함.

- 해당 평가 항목은 방문진료 시 안정적이고 조용한 환경에서 평가자와 연구대상자가 원활히 소통할 수 있도록 준비된 공간에서 시행되며, 평가자가 항목별로 직접 관찰 또는 면담을 통해 기재함.

- 방문점검기록지는 각 직역(의사, 간호사, 사회복지사)에 따라 방문진료시 해당 전문 인력이 직접 작성함.

- 응답자의 언어·청력·인지기능 등의 문제로 인해 본인이 자가응답을 할 수 없는 경우, 법정 대리인 또는 돌봄 제공자의 대리응답을 허용하며, 이는 해당 사례에 대해 연구자가 사전에 판단하여 결정함.

• 국가승인통계인 장애인 실태조사의 대리 응답은 1. 부모 2. 배우자 3. 형제자매 4. 자녀 5. 이웃이나 친척 6. 활동지원사, 요양보호사 등 7. 기관종사자에게 허용됨.

• 본 연구에서는 대상자의 상태를 정확하게 파악할 수 있도록 더 엄격한 선행 연구(조미희, 2024)의 기준을 적용함.

• 본 연구의 대리 응답자는 법정 대리인 혹은 돌봄 제공자로, 돌봄 제공자의 경우 하루 평균 3시간 이상 실질적인 돌봄을 수행하는 자로 한정하며, 다음 범주에 해당하는 자를 포함함: (1) 가족 보호자, (2) 요양보호사, (3) 장애인 활동지원사

○ 만족도 조사

- 환자 만족도 조사(CSQ)는 객관적 평가 확보를 위해, 연구참여기관과 독립적인 소속의 송수민 연구자가 전담하여 실시함.

- 조사는 연구기간 중 기저조사와 추적조사에 모두 참여하였으며, 최소 1회 이상의 재택의료 진료를 받은 환자 본인을 대상으로 함.

- 자가응답이 곤란한 경우, 본 조사는 대리응답을 허용하지 않으며 해당 시점의 조사는 제외함.

- 조사는 방문 대면 조사를 원칙으로 하며, 대면 조사가 불가능한 경우에는 유선 인터뷰로 대체할 수 있음. 유선조사의 경우에도 동일한 조사 지침과 문항을 적용하고, 응답자의 신원을 충분히 확인한 후 진행함.

**3) 수집 및 관리 방법**

○ 평가자 교육 방법

- 모든 평가의 일관성을 확보하기 위해 표준운영지침서를 제작하고, 연구 시작 전 모든 평가자를 대상으로 워크숍을 진행함.

- 연구 초기에 2명의 평가자가 동일한 환자 몇 명을 평가하여 그 결과가 얼마나 일치하는지 보는 평가자 간 신뢰도를 검증하고, 그 결과가 낮을 경우 재교육을 실시함.

○ 자료수집 방법

- 연구진, 의무기록·설문·전산자료 조사 담당자와의 협의를 통해 자료 수집 방법을 확정함.

- 증례기록지의 경우 종이 형태로 작성하여, 원본은 연구참여기관에 보관하며, 사본은 동신대학교 배예린 연구원에게 전송함.

- 포괄평가 기록지와 방문점검 기록지(의사, 간호사, 사회복지사)의 경우 원본은 연구 참여기관에 보관하며, 증례기록지에서 수집 예정인 대상자의 개인 정보(성명, 생년월일, 성별, 주소) 외의 개인 정보는 삭제한 상태로, 사본을 동신대학교 배예린 연구원에게 전송함.

- 전송된 데이터는 클리닝 절차를 거쳐 한의약혁신기술개발사업단이 제공하는 전자증례기록지(eCRF; iClick)에서 체계적으로 관리하여 연구 투명성을 확보함.

**13. 효과 평가 기준 및 방법**

**1) 재택의료센터 및 환자의 특성**

○ 포괄평가 기록지, 방문점검 및 업무 기록지에 포함되어있는 재택의료센터의 진료 정보와 환자의 일반적인 특성에 대해 분석할 예정임.

- 한의과 재택의료센터 환자의 기초 특성을 기술적으로 분석함.

- 이러한 분석 결과는 향후 효과 해석의 보조적 근거로 활용함.

**2) Primary outcome**

○ K-ADL

- 기능 상태를 나타내는 K-ADL 점수를 주요 평가지표로 설정하였음.

- K-ADL 점수는 치료 전(기저조사)과 치료 후(추적조사)의 변화량을 비교 평가함.

**3) Secondary outcome**

○ K-FRAIL, NRS는 점수 변화량을 통해 평가함.

○ CSQ는 레지스트리 참여 후 평가 점수를 기술적으로 평가함.

○ 포괄평가 기록지, 방문점검 및 업무 기록지

- 레지스트리 기반 분석 특성상 사전에 특정 통계적 가설을 고정적으로 설정하기보다는, 연구 기간 중 도출되는 질문에 따라 분석 범위와 방법을 단계적으로 확장할 계획임.

- 포괄평가 기록지 항목은 변수 특성에 따라 점수형 변수와 사건형 변수로 구분되며, 이에 따라 본 연구에서 제시한 효과 평가 기준을 기준으로 항목별 분석을 수행할 예정임.

**14. 예측 부작용 및 주의 사항과 조치**

**1) 관찰연구 참여로 인한 추가 부작용**

○ 연구 참여로 인해 발생 가능한 추가적인 부작용은 없을 것으로 예상됨.

- 본 연구는 재택의료센터의 일상 진료 환경 내에서 이루어지는 관찰연구이며, 별도의 침습적 검사나 연구 목적의 추가 치료는 포함되지 않음.

- 이러한 연구 특성을 고려하여, 본 연구에서는 중대한 이상반응만을 수집 대상으로 하며, 다음의 경우를 중대한 이상반응으로 정의함.

• 사망

• 30일을 초과하는 장기 입원이나 시설입소가 필요하여 재택 치료가 불가능한 상황

**2) 이상반응 발생 시 조치 및 보고**

○ 연구진은 방문 진료 시 또는 대상자나 보호자의 연락을 통해 중대한 이상반응 발생 여부를 확인하며, 발생 인지 시 다음의 절차에 따라 신속하게 조치하고 보고함.

- 입원 및 시설 입소가 필요한 경우

• 상태 평가 및 권고: 연구참여기관의 연구 한의사는 평소 방문 진료 시 대상자의 활력징후, 주관적 증상, 임상적 관찰 및 평가 등을 통해 대상자의 상태를 종합적으로 평가함. 평가 결과, 재택의료의 범위를 넘어선 집중 치료나 돌봄이 필요하다고 판단될 경우, 대상자와 보호자에게 입원 또는 시설 입소를 적극적으로 권유함.

• 의료기관 연계: 입원 또는 입소가 결정되면, 연구 한의사는 대상자의 상태에 적합한 상급 의료기관이나 요양시설에 대한 정보를 제공하고, 필요한 경우 진료의뢰서를 발급하는 등 원활한 연계가 이루어지도록 지원함.

• 연구 참여 중단 처리: 30일을 초과하는 장기 입원 또는 시설 입소로 인해 더 이상 본 연구의 추적 관찰이 불가능하게 된 시점에서 해당 대상자는 연구 참여 중단으로 처리되며, 사유와 경과를 증례기록서에 상세히 기록함.

- 재택의료 중 사망이 발생한 경우

• 발생 인지 및 조치: 보호자의 연락이나 연구 한의사의 직접 관찰 등을 통해 대상자의 사망을 확인한 즉시, 연구책임자는 임종 돌봄 임상진료지침 및 관련 법규에 따라 신속히 조치함.

• 사망진단서 발급: 필요한 경우 연구 한의사는 관련 법규에 따라 사망진단서를 발급하고, 유가족에게 애도를 표하며 필요한 행정 절차를 안내함.

• 기록 및 연구 처리: 사망 사실과 시점, 경위 등을 확인하여 공식적으로 진료기록부에 기록함. 사망으로 인해 더 이상 본 연구의 추적 관찰이 불가능하게 된 시점에서 해당 대상자는 연구 참여 중단으로 처리되며, 증례기록서에 상세히 기록함.

- 기관생명윤리위원회 보고

• 연구 참여로 인한 중대한 이상반응은 없을 것으로 예상되나, 고령자를 대상으로 한 연구이므로 연구 진행 중에 사망, 입원 및 시설입소가 발생할 것으로 예상됨.

• 따라서, 기관별로 지속심의 시 전체 기관의 누적 사망, 입원 및 시설입소 건을 모아서 함께 보고함.

**15. 자료 분석과 통계적 방법**

**1) 재택의료센터 및 환자의 특성**

○ 분석에 앞서 환자 및 재택의료센터 특성에 대해 횡단적으로 기술통계를 산출함.

- 연속형 변수는 평균 ± 표준편차 또는 중앙값(사분위수)으로, 범주형 변수는 빈도(%)로 제시함.

○ 중재 효과에 영향을 미치는 요인을 파악하기 위해, 주요 환자 및 기관 특성 변수를 독립변수로 포함한 다변량 회귀모형을 활용함.

- 시간과 집단 간 상호작용 외에도 개별 특성과의 상호작용항을 포함한 모형을 구성하여, 특정 요인에 따라 중재 효과가 달라지는지를 평가함.

**2) Primary outcome: K-ADL**

○ 본 연구는 대조군이 없는 단일군 관찰연구로서 연구 전후의 점수를 비교함.

- Paired t-test (혹은 Wilcoxon signed-rank test)를 수행함.

- 통계적 유의수준은 0.05 미만으로 설정함.

| 가설 설정:  귀무가설(H₀): 기저조사 대비 추적조사의 K-ADL 점수가 변화 없이 동일하다.  대립가설(H₁): 기저조사 대비 추적조사의 K-ADL 점수가 같지 않다. |
| --- |

**3) Secondary outcome**

○ K-FRAIL: Paired t-test (혹은 Wilcoxon signed-rank test)를 수행함.

○ NRS: Repeated Measures ANOVA (혹은 Friedman Test)를 수행함.

○ CSQ: 연구 종료 시점에만 측정하므로, 기술통계를 수행함.

○ 통계적 유의수준은 0.05 미만으로 설정함.

**4) 포괄평가 기록지, 방문점검 및 업무 기록지**

○ 포괄평가 기록지와 방문점검 및 업무 기록지의 데이터는 다양한 영역에 걸친 연속형 변수 및 사건형 변수로 구성되어 있으며, 각 변수의 속성에 따라 적절한 통계분석 방법을 적용할 예정임.

- 시간 경과에 따른 변화를 분석하기 위한 반복 측정 자료는 측정 횟수에 따라 다른 방법을 적용함.

- 측정 시점이 2회인 경우, 연속형 변수의 변화는 Paired t-test, 범주형 변수는 McNemar’s test로 분석함.

- 만약 측정 시점이 3회 이상이라면, 연속형 변수는 Repeated Measures ANOVA나 Linear Mixed Model을, 범주형 변수는 Cochran’s Q test 또는 Generalized Estimating Equations을 사용하여 분석할 계획임.

- 모든 통계 분석의 유의수준은 0.05 미만으로 설정함.

**5) 치료반응군 분석**

○ 치료 전후 K-ADL 점수 변화량이 사전에 정의된 MCID(Minimum clinically important difference) 값 이상인 경우를 '임상적으로 유의한 호전군'으로 정의하고, 이에 영향을 미치는 예측 요인을 탐색함.

- 성별, 연령, 심혈관질환 여부, 당뇨 여부, 고혈압 여부, 고지질혈증 여부, 치료 전 K-FRAIL 점수, 치료 전 Five-times chair and stand test (5CST) 점수, 치료 전 Timed up and go test (TUG test) 점수, 치료 전 10계단 올라가기 능력 점수 등을 보정한 다변량 로지스틱 회귀분석을 시행함.

**6) 결측치 처리 방법**

○ 본 연구에서 예상되는 결측 데이터는 통계 분석의 편향을 최소화하고 결과의 신뢰도를 확보하기 위해 다중 대체법(Multiple Imputation, MI)을 적용하여 처리함.

- 대체 방법: 연쇄방정식을 이용한 다변량 대체 알고리즘을 사용하여 결측값을 예측하고, 총 20개의 가상적 완전 데이터셋을 생성함.

- 대체 모델 변수: 대체 모델의 정확성을 높이기 위해, 주요 결과 변수(K-ADL)와 주요 예측 변수(연령, 성별 등)를 모두 포함함.

- 분석 및 통합: 생성된 20개의 데이터셋 각각에 대해 주 분석을 독립적으로 시행한 후, 루빈의 규칙에 따라 그 결과들을 하나의 최종 추정치로 통합함,

**16. 동의 철회 및 중도 탈락**

**1) 중도 탈락 기준**

○ 연구 도중 다음과 같은 경우에 해당될 경우, 연구대상자는 중도 탈락할 수 있음.

- 연구대상자가 연구 참여 도중 자발적으로 참가 동의를 철회한 경우

- 연구 진행 중 연구대상자 및 법정대리인의 동의능력이 결여된 경우

- 연구대상자가 선정기준에 부합하지 않거나 제외기준에 해당하는 것이 확인된 경우

- 중대한 이상사례가 발생한 경우

- 그 외, 연구 책임자 또는 담당자의 판단에 따라 연구 지속이 대상자에게 적절하지 않다고 판단되는 경우

○ 중도 탈락 시점까지 수집된 자료는 연구에 포함되며, 연구동의서에 서명할 당시 또는 연구 진행 중, 탈락 이후라도 개인정보 및 자료 활용에 대해 명시적으로 동의를 철회한 경우에는 해당 연구자료는 분석에서 제외함.

○ 본 연구에서 중도탈락한 대상자는 이후 다시 연구에 재참여하지 않음.

**2) 연구중지 기준**

○ 연구가 연구대상자의 안전과 복지에 중대한 위험을 초래한다고 판단되는 경우, 연구는 조기에 중단될 수 있음

**3) 동의 철회**

○ 연구대상자는 본 연구에 자발적으로 참여에 동의하였더라도, 연구 중 언제든지 동의를 철회할 수 있으며, 동의 철회로 인해 어떠한 불이익도 받지 않음.

**17. 연구대상자의 위험과 이익**

**1) 예상되는 위험**

○ 본 연구는 기존 진료 환경에서 제공되는 표준적인 재택의료서비스와 평가 설문조사를 기반으로 하며, 연구 참여로 인해 추가적인 시술이나 침습적 중재가 시행되지 않으므로, 예상되는 위험은 없음.

**2) 기대되는 이익**

○ 연구대상자에게는 다음과 같은 직접적 또는 간접적 이익이 제공될 수 있음:

- 정기적인 포괄평가 및 상태 모니터링을 통해 환자의 건강 상태에 대한 지속적인 확인 및 관리가 이루어짐

- 중재 이후 일상생활 수행능력, 통증 등 여러 영역에서 개선 가능성이 있음

- 본인의 진료 정보가 향후 돌봄 정책 개선의 근거자료로 활용됨으로써, 간접적으로 공공 보건의료 향상에 기여할 수 있음

**18. 연구 참여에 따른 보상**

○ 연구 참여에 따른 보상으로 2만원 상당의 위생용품과 한방 파스를 1회 제공할 예정임.

**19. 연구대상자 안전대책 및 개인 정보 보호 대책**

**1) 일상적인 진료 및 환자관리를 위한 정보 수집**

○ 본 연구에서는 연구대상자의 진료 및 평가를 위해 다음과 같은 정보를 수집함.

- 개인정보: 이름, 성별, 생년월일, 주소

- 민감정보: 포괄평가지 및 방문점검 기록지 내 환자 특성 정보, 각종 설문 결과

- 수집하는 개인정보 이외의 포괄평가지 내 대상자 정보는 수집하지 않음.

| ※ 수집하는 포괄평가지 및 방문점검 기록지 정보  ‧ 포괄평가지: 방문일, 기관정보, 대상자 정보(성명, 생년월일, 성별, 장기요양등급, 주소), 건강/질병 상태(해결 요청 주요 문제, 치료중인 동반 질환, 투약 내용 등), 신체상태(보행능력 및 근력 검사, 영양상태 및 식사행위 등), 정신상태(인지기능, 우울 등), 자립생활 가능성(의식수준, 신체기능, 인지기능 등), 의료처치 및 건강관리 필요 항목, 사회/환경 평가(일상생활 도움 필요 여부, 경제상황, 사회관계 등), 서비스 이용현황(의료서비스, 장기요양서비스 등)  ‧ 방문점검 기록지(의사): 기관정보, 대상자 정보(성명, 생년월일, 성별, 장기요양등급, 주소), 서비스 제공시간, 방문사유, 신체기능 자립성, 인지기능 자립성, 진찰 및 상담 내용, 침습적 처치 내용, 간호지시, 향후계획  ‧ 방문점검 기록지(간호사): 기관정보, 대상자 정보(성명, 생년월일, 성별, 장기요양등급, 주소), 방문 사유, 서비스 제공시간, 동행자, 방문내용(투약관리 포함), 건강상태, 혈압/맥박, 체온/혈당, 체중 변화, 섬망, 낙상, 소변/대변실금, 향후계획  ‧ 방문점검 기록지(사회복지사): 기관정보, 대상자 정보(성명, 생년월일, 성별, 장기요양등급, 주소), 상담 내용, 지역사회연계 |
| --- |

○ 이 모든 정보는 일상적인 진료 및 환자관리 과정에서 통상적으로 수집되는 항목이며, 본 연구의 수집 범위는 이를 초과하지 않음.

○ '연구 참여 동의서'와는 별도로, '개인정보 제3자 제공 및 2차 연구 이용 동의서'를 연구대상자에게 제공할 예정임.

- 연구대상자가 이 별도 동의서에 서명하는 경우에 한하여, 수집된 연구 정보를 제3자에게 제공하고 2차 연구 목적으로 이용하도록 함.

- 연구대상자는 '개인정보 제3자 제공 및 2차 연구 이용 동의서'에 동의하지 않더라도, '연구 참여 동의서'에 동의한 것만으로도 본 환자등록연구에 참여할 수 있음.

- '개인정보 제3자 제공 및 2차 연구 이용 동의서'에 동의할 경우: 연구대상자의 정보는 향후 한의약진흥원에 제공되며, 국가 공공기관(건강보험심사평가원, 통계청, 국민건강보험공단, 질병관리본부, 국립암센터 등)의 데이터베이스와 연계·결합되어 다른 연구에 활용될 수 있음.

- '개인정보 제3자 제공 및 2차 연구 이용 동의서'에 동의하지 않을 경우: 연구대상자의 정보는 한의약진흥원에 제공되지 않으며 본 연구의 목적으로만 사용됨.

**2) 개인정보의 비밀 유지**

○ 대상자의 신원을 파악할 수 있는 기록은 비밀로 보장될 것이며, 관찰연구의 결과가 출판될 경우에도 대상자의 신원을 비밀상태로 유지함.

- 수집된 환자의 개인정보가 포함된 모든 연구자료는 연구책임자 및 공동연구자만이 이를 열람할 수 있음.

- 연구자는 본 관찰연구의 계약이 체결됨으로써 연구의뢰자 또는 점검자가 연구대상자의 차트와 증례기록서를 검증하기 위하여 해당 문서를 검토하거나 복사할 수도 있음을 숙지하여야 함.

**3) 연구자료의 보관 방법 및 장소**

○ 관련 자료는 허가받은 사람만이 접근할 수 있도록 시건장치를 달아서 관련자 이외의 사람의 접근을 막을 수 있는 곳에 보관함.

- 연구진 진료실 시건장치가 달린 캐비닛과 암호로 접근이 제한되는 컴퓨터, 분석자 및 평가자의 연구실에 암호로 접근이 제한되는 컴퓨터에 보관하여 연구책임자, 공동연구자만 접근이 가능하도록 함.

**4) 자료 보관 기간**

○ 생명윤리 및 안전에 관한 법률 시행규칙 제15조에 따라 연구자료 (기관위원회 심의결과, 동의서, 개인정보수집/이용.제공현황, 연구종료보고서)는 연구종료 후 최소 3년간 보관하며, 분석자 및 평가자에게 전달된 데이터 또한 연구가 종료된 시점부터 최소 3년간 보관함.

**5) 자료 폐기 방법**

○ 전자문서를 포함한 모든 연구자료(계획서, IRB 심의결과, 연구종료보고서 등)는 보관 기간 이후 문서파쇄기 이용 또는 전자 파일 삭제 방식으로 영구 폐기함.

○ iClick을 통해 한의약혁신기술개발사업단에 제공된 정보는 연구종료 10년 후까지 보관 후 삭제함.

**20. 참고문헌**

- 통계청. (2023). 장래인구추계. 통계청. https://kostat.go.kr/
- 대한민국 정책브리핑. (2018, 11월 19일). 지역사회 통합 돌봄 기본계획(1단계: 노인 커뮤니티케어) 발표 [보도자료]. 보건복지부.
- 대한민국 정책브리핑. (2021, 11월 17일). 지역사회 통합 돌봄(커뮤니티 케어). https://www.korea.kr/special/policyCurationView.do?newsId=148866645#L1
- 노인장기요양보험법 시행령, 제2조. (2023).
- 국민건강보험공단. (2023). 노인장기요양보험 통계연보. 국민건강보험공단.
- 대한노인의학세부전문의관리위원회. (2023). 노인의학 세부전문의. 의학출판사.
- 김창오. (2019, 10월). 노쇠와 노인증후군. 대한내과학회 추계학술발표논문집, 97(2), 716–718. 대한내과학회.
- Won, C. W., Yoo, H. J., Yu, S. H., Kim, C. O., Dumlao, L. C. I., Dewiasty, E., ... & Prakash, O. (2013). Lists of geriatric syndromes in the Asian-Pacific geriatric societies. European Geriatric Medicine, 4(5), 335-338.
- 구현지, & 방준석. (2018). 노인증후군의 이해. 대한약국학회지, 4(1), 41-50.
- 한국한의약진흥원. (2024). 한의 장기요양 재택의료센터 안내서 개발 연구. 한국한의약진흥원.
- 보건복지부. (2025). 장기요양 재택의료센터 시범사업 지침(한의원). https://www.mohw.go.kr/board.es?mid=a10501010100&bid=0003&act=view&list_no=1484242&tag=&nPage=1
- Allen, A., Patrick, H., Ruof, J., Buchberger, B., Varela-Lema, L., Kirschner, J., ... & Guilhaume, C. (2022). Development and pilot test of the registry evaluation and quality standards tool: an information technology–based tool to support and review registries. Value in Health, 25(8), 1390-1398.
- Asher, A. L., Parker, S. L., Rolston, J. D., Selden, N. R., & McGirt, M. J. (2015). Using clinical registries to improve the quality of neurosurgical care. Neurosurgery Clinics of North America, 26(2), 253-63.
- Gliklich, R. E., Dreyer, N. A., & Leavy, M. B. (Eds.). (2014). Registries for evaluating patient outcomes: a user’s guide.
- World Health Organization. (2001). International classification of functioning, disability and health (ICF). WHO.
- 보건복지부. (2018). 장기요양등급판정기준에 관한 고시, 제2조(장기요양인정점수 산정방법). 보건복지부.
- Fried, L. P., Ferrucci, L., Darer, J., Williamson, J. D., & Anderson, G. (2004). Untangling the concepts of disability, frailty, and comorbidity: implications for improved targeting and care. The journals of Gerontology Series A: Biological sciences and Medical sciences, 59(3), M255-M263.
- World Health Organization. (2025). ICD-11 for mortality and morbidity statistics. https://icd.who.int
- 이영진. (2006). 만성 통증의 발생기전과 중재적 신경-근 자극요법. Korean Journal of Family Medicine, 27(5), 341-351.
- Samuelsen, P. J., Svendsen, K., Wilsgaard, T., Stubhaug, A., Nielsen, C. S., & Eggen, A. E. (2016). Persistent analgesic use and the association with chronic pain and other risk factors in the population—a longitudinal study from the Tromsø Study and the Norwegian Prescription Database. European journal of clinical pharmacology, 72(8), 977-985.
- Clark, J. D. (2002). Chronic pain prevalence and analgesic prescribing in a general medical population. Journal of pain and symptom management, 23(2), 131-137.
- 원장원, 등. (2002). 한국형 일상생활활동 측정도구(K-ADL)와 한국형 도구적 일상생활활동 측정도구(K-IADL)의 개발. 대한노인병학회지, 6(2), 107–120.
- 유효선. (2021). 일차의료에서 활용 가능한 노쇠 임상진료지침. Korean Journal of Family Practice, 11(4), 229–236.
- Fried, L. P., Tangen, C. M., Walston, J., Newman, A. B., Hirsch, C., Gottdiener, J., ... & McBurnie, M. A. (2001). Frailty in older adults: evidence for a phenotype. The Journals of Gerontology Series A: Biological Sciences and Medical Sciences, 56(3), M146-M157.
- Williams, A. C. D. C., Davies, H. T. O., & Chadury, Y. (2000). Simple pain rating scales hide complex idiosyncratic meanings. Pain, 85(3), 457-463.
- 조미희, 신유미, 최보경, 최은희, 이혜연, 이지환, ... & 김창오. (2024). 재택의료에 대한 환자 만족도와 서비스 질 평가 연구. 한국노년학, 44(4), 509-521.
