# Supplementary material for: The Korean Medicine HOme Medical care for the Elderly (K-HOME) registry: A study protocol for a multicenter registry on aging in place and functional recovery
Source: PLoS One. 2026 Apr 30;21(4):e0347574. doi: 10.1371/journal.pone.0347574 (PMC13132427; doi:10.1371/journal.pone.0347574)
Supplement: S3 File — (DOCX) [file pone.0347574.s003.docx]

**[Recommendation Form No. 38] Research Protocol (For Human Subject Research / Experimental Study) ver4.0**

*Public Institutional Review Board (IRB) Designated by the Ministry of Health and Welfare*

**Clinical Study Protocol**

**(For Human Subject Research**

**/ Experimental Study)**

ver1.4

Title of Research : Patient Registry Study of Elderly Patients

with Functional Decline at Home Medical Centers

Researcher's Affiliation and Name

Jungdong Korean Medicine Clinic, Beom-seok Kim

Geonganghanmaeul Korean Medicine Clinic, Kwon-hee Kim

Dongbang Shintong Bubu Korean Medicine Clinic, Ho-yeol Bang

Haemalgeun Korean Medicine Clinic, Chang-hoon Kim

Kim Jeong-cheol Korean Medicine Clinic, Jeong-cheol Kim

Seohwa Korean Medicine Clinic, Hee-jun Sim

College of Korean Medicine, Dongshin University, Assistant Professor Dong-su Kim

College of Korean Medicine, Dongshin University, Graduate Student Han-bit Jin

College of Korean Medicine, Dongshin University, Graduate Student Eun-ji Ahn

College of Korean Medicine, Dongshin University, Researcher Ye-rin Bae

College of Korean Medicine, Wonkwang University, Graduate Student Hyeong-seon Jeon

Doctors of Korean Medicine for Health Rights, Soo-min Song

**▣ Protocol Amendment/Revision History**

| **No** | **Version No.** | **Version Date** | **Key Contents** |
| --- | --- | --- | --- |
| 1 | 1.0 | 2023. 05. 20. | First Edition of the Clinical Study Protocol |
| 2 | 1.1 | 2023. 08. 04. | Revision to Ver. 1.1 incorporating amendments from other institutions prior to IRB submission. |
| 3 | 1.2 | 2025. 09. 11. | Revision to Ver. 1.2 incorporating amendments from other institutions prior to IRB submission. |
| 4 | 1.3 | 2025. 10. 13 | Revision to Ver. 1.3 incorporating feedback from the Public IRB, and subsequent IRB approval. |
| 5 | 1.4 | 2025. 11. 12 | Amendment to Ver. 1.4 incorporating revisions from other institutions, and subsequent IRB approval. |

1. Research Background

1) Current status of Domestic and International R&D Projects

A. Socioeconomic Background

○ The domestic population is experiencing an increase in life expectancy and morbidity during old age, accompanying the deepening of the aging process.

- The proportion of the population aged 65 and over in South Korea is 19.2% as of 2024. This proportion is projected to reach 20.3% in 2025, which will mark the country's entry into a super-aged society.


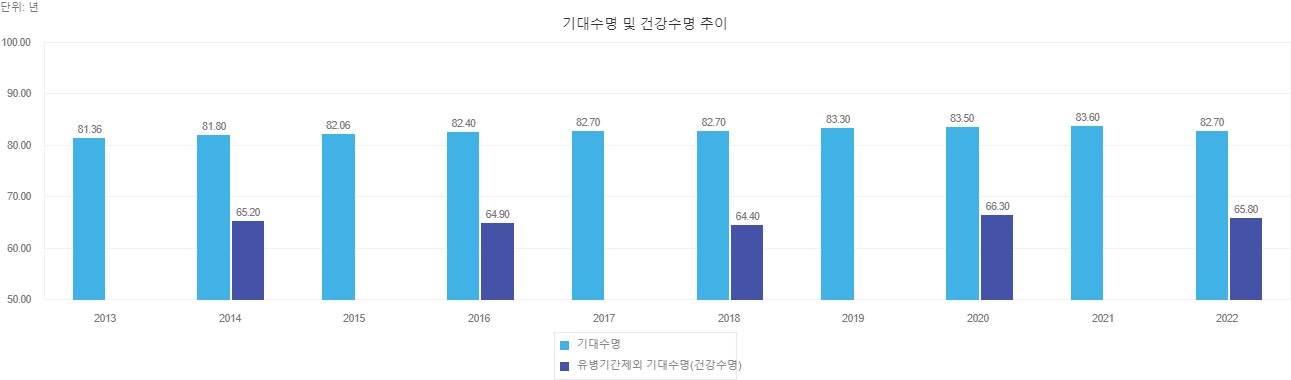


○ The share of the population aged 65 and over in Korean medicine expenditure increased from 32% in 2014 to 37% in 2021.


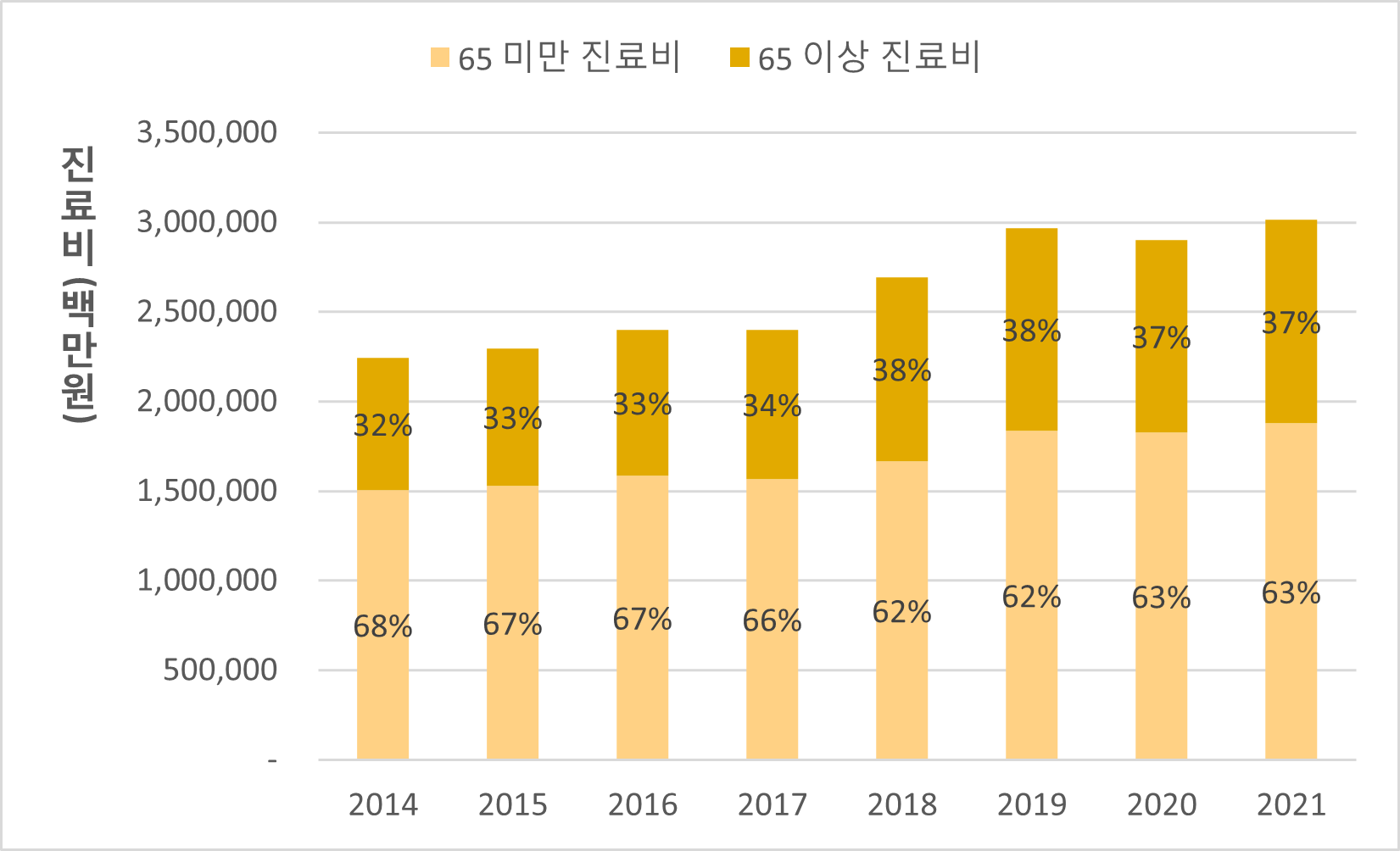


○ The government has established comprehensive management policies for the elderly to meet the demand for elderly care and alleviate the financial burden on the healthcare system.

- In 2018, the government announced the Integrated Community Care Basic Plan and implemented the Integrated Community Care Pilot Project for two years starting in June 2019 across 16 local governments (cities, counties, and districts).
A locally-led social service policy that integrates support for healthcare, long-term care, community care, and independent living.

- To address the increasing demand for healthcare and care services, the ‘Integrated Support Pilot Project for Elderly Healthcare and Care (July 2023 – December 2025)’ is being implemented to provide the elderly with integrated and linked support for healthcare and care services in their residential communities.

- ‘Pilot Project for Home medical centers for Long-Term Care Recipients’ targets Long-Term Care Insurance Grade 1-4 beneficiaries whom physicians determine require home medical care due to limited mobility. Physicians, nurses, and social workers periodically visit their homes to provide visiting medical care, nursing, and community care linkage.

B. Definition and Current Status of Frail Older Adults

○ The government has been implementing the Long-Term Care Insurance System for Older Adults (hereinafter, Long-Term Care Insurance) since 2008 to provide care coverage for older adults who require long-term care due to functional decline.

- Beneficiaries of the Long-Term Care Insurance are individuals who have geriatric diseases and whose physical or cognitive functional decline makes it difficult for them to perform daily activities independently (for a period of six months or longer). They can be regarded as the representative group of frail older adults.

| **< Definition of Eligibility for Long-Term Care Insurance >**  ∙ Older adults aged 65 or over, or individuals under the age of 65 who have geriatric diseases prescribed by Presidential Decree, such as dementia or cerebrovascular disease  ∙ Older adults who have difficulty performing daily activities independently due to aging or geriatric diseases  ∙ The Long-Term Care Insurance System provides these individuals with long-term care benefits, such as assistance with physical activities or household chores.  Source: Reorganized contents from Article 2 of the Long-Term Care Insurance Act for Older Adults |
| --- |

Beneficiaries are classified into grades 1 through 5 and the Cognitive Support Grade based on their physical and mental functional status. Grades 1 and 2, and Grades 3 and 4, as well as Grade 5 and the Cognitive Support Grade, are limited to those with dementia (dementia corresponding to geriatric diseases as prescribed in Article 2 of the Enforcement Decree of the Long-Term Care Insurance Act for Older Adults).

<Table 3> Status of Long-Term Care Grade Determinations

| Category | | Total | Approved Applicants | | | | | | | Individuals Not Classified | | | |
| --- | --- | --- | --- | --- | --- | --- | --- | --- | --- | --- | --- | --- | --- |
|  |  |  | Subtotal | Grade 1 | Grade 2 | Grade 3 | Grade 4 | Grade 5 | Cognitive Support Grade | Subtotal | Out-of-Grade  A | Out-of-Grade  B | Out-of-Grade  C |
| 2024  (Q3) | Count | 1,282,878 | 1,147,469 | 54,519 | 98,989 | 307,216 | 527,096 | 132,393 | 27,256 | 135,409 | 73,372 | 43,758 | 18,279 |
|  | Percentage | 100% | 89.40% | 4.20% | 7.70% | 23.90% | 41.10% | 10.30% | 2.10% | 10.60% | 5.70% | 3.40% | 1.40% |
| 2023 | Count | 1,238,495 | 1,097,913 | 52,913 | 98,015 | 297,796 | 499,584 | 123,971 | 25,634 | 140,582 | 75,552 | 47,410 | 17,620 |
|  | Percentage | 100% | 88.60% | 4.30% | 7.90% | 24.00% | 40.30% | 10.00% | 2.10% | 11.40% | 6.10% | 3.80% | 1.40% |
| 2022 | Count | 1,160,850 | 1,019,130 | 49,946 | 94,233 | 278,520 | 459,316 | 113,842 | 23,273 | 141,720 | 74,878 | 50,385 | 16,457 |
|  | Percentage | 100% | 87.80% | 4.30% | 8.10% | 24.00% | 39.60% | 9.80% | 2.00% | 12.20% | 6.50% | 4.30% | 1.40% |
| 2021 | Count | 1,097,462 | 953,511 | 47,800 | 92,461 | 261,047 | 423,595 | 106,107 | 22,501 | 143,951 | 74,838 | 53,700 | 15,413 |
|  | Percentage | 100% | 86.90% | 4.40% | 8.40% | 23.80% | 38.60% | 9.70% | 2.10% | 13.10% | 6.80% | 4.90% | 1.40% |
| 2020 | Count | 1,007,423 | 857,984 | 43,040 | 86,998 | 238,697 | 378,126 | 91,960 | 19,163 | 149,439 | 76,481 | 58,659 | 14,299 |
|  | Percentage | 100% | 85.20% | 4.30% | 8.60% | 23.70% | 37.50% | 9.10% | 1.90% | 14.80% | 7.60% | 5.80% | 1.40% |
|  | | | | | | | | | | | | | |

Source: National Health Insurance Service website. Long-Term Care Insurance Business Performance, Status of Long-Term Care Grade Determinations. September 30, 2024.

- According to the National Health Insurance Service’s 2023 Statistical Yearbook of Long-Term Care Insurance for Older Adults, the total number of approved applicants of Long-Term Care Insurance was 1,097,913 as of the end of December 2023. This figure accounts for 11.1% of the population aged 65 and over and has shown a continuous increasing trend annually over the past five years.

○ As changes resulting from physiological aging and the cumulative effect of diseases occur with increasing age, geriatric diseases present vague and complex clinical patterns. This characteristic is referred to as Geriatric Syndrome.

- Frail older adults have a relatively high risk of Geriatric Syndrome, and once the vicious cycle of Geriatric Syndrome begins, it is extremely difficult to restore their function to the previous level.

- According to a study conducted in 2013, focusing on the views of geriatric specialists in the Asia-Pacific region, Geriatric Syndrome includes conditions such as frailty, sarcopenia, limited mobility, gait disturbance, pressure ulcers, and dementia.

C. Current Status of Korean Medicine's Participation in Integrated Care and Home medical centers

○ Since the launch of the Integrated Community Care project in 2019, practitioners of Traditional Korean Medicine have actively participated in integrated care and home medical center projects and have achieved significant results in managing the health of frail older adults.

- Korean Medicine care projects have continuously expanded, covering 13 regions in 2021 and 20 regions in 2022 under the Integrated Community Care project, and further increasing to 25 regions in 2023 when transitioning to the Integrated Support Pilot Project for Elderly Healthcare and Care. Regular monitoring is also being carried out, including the discovery of best practices and the sharing of outcomes annually.

- Reflecting the demand for Korean Medicine care, the Long-Term Care Home medical center Pilot Project saw Korean Medicine clinics increase their participation from 3 out of 28 medical institutions in the first phase to 24 out of 95 in the second phase.

| 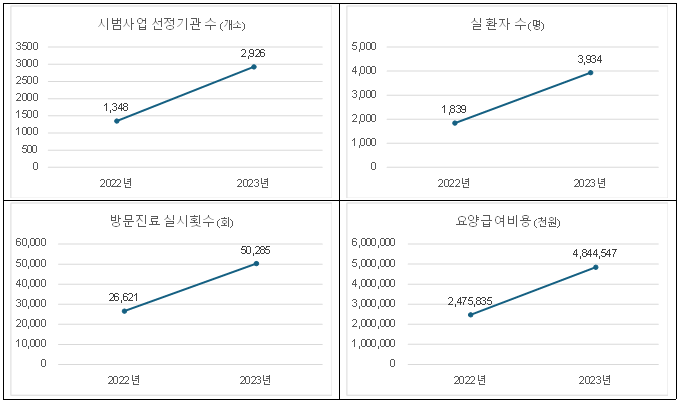  **<** **Pilot Project for Korean Medicine Primary Care Home Visit Fee (2022–2023) >** | 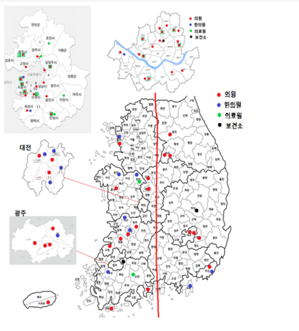  **<** **Current Status of Korean Medicine Long-Term Care Home medical centers >** |
| --- | --- |

○ Long-Term Care Insurance beneficiaries can receive Korean Medicine care services through Korean Medicine Long-Term Care Home medical centers (hereinafter, KM Home medical centers) and other means.

- The research team investigated the current status of medical treatment and disease characteristics of frail older adults utilizing Korean Medicine through a survey conducted among Korean Medicine practitioners who perform home visits under the KM Home medical center Project, as part of the preceding study, "A Study on the Development of a Guideline for Korean Medicine Long-Term Care Home medical centers" (National Institute for Korean Medicine Development, 2024)

- In the survey conducted on 765 Long-Term Care Insurance beneficiaries registered at 22 KM Home medical centers, the subjects mainly requested home visits by a Korean Medicine practitioner when they had issues related to chronic disease/pain management, physical function rehabilitation, and geriatric syndrome.

- The results of the multiple-choice survey on the symptoms and management actually handled by Korean Medicine practitioners during home visits showed that 'musculoskeletal pain relief' was the most common at 32.9%, followed by 'monitoring and management of metabolic diseases such as hypertension, diabetes, and dyslipidemia' at 13.2%, and 'physical function rehabilitation, including paralysis and contracture management' at 11.7%.

- It was also confirmed that Korean Medicine practitioners provide treatment and management for various diseases and symptoms of the beneficiaries, including 'management of urinary incontinence and urination' (6.7%), 'relief of psychiatric disorders such as dementia, sleep disorders, depression, and anxiety' (6.2%), and 'management of skin diseases such as pressure ulcers' (3.6%).

2) Necessity of the Study

A. Necessity of the Study

○ The government plans to launch the Integrated Support Project for Older Adults' Healthcare and Care as a formal program in 2026, but the Korean Medicine sector currently lacks sufficient research in this area.

- Despite the active participation of the Korean Medicine sector, systematic studies evaluating the outcomes of KM care are lacking. Furthermore, despite high participation on the ground, there is insufficient policy evidence to directly confirm the role of Korean Medicine.

- Therefore, there is a need to establish the foundation for Korean Medicine's participation by comprehensively presenting the project outcomes of Korean Medicine care.

○ Systematic evaluation of the Korean Medicine clinic home medical center pilot project is necessary to establish a policy basis.

- Prior to the implementation of the main project, the foundation for the development and dissemination of a standardized Korean Medicine home medical care model must be laid by evaluating the characteristics and effectiveness of the Korean Medicine approach from various angles.

- There is a need to quantitatively assess the overall effectiveness of the service by jointly evaluating Activities of Daily Living (ADLs), patient health indicators, and satisfaction.

2. Research Objectives

1) Research Objectives

○ Primary Objective

- To identify changes in Activities of Daily Living (ADLs) indicators among frail older adults treated at Korean Medicine Home medical centers.

○ Secondary Objectives

- To identify changes in frailty and chronic pain indicators among frail older adults treated at Korean Medicine Home medical centers.

- To analyze the predictors (such as patient characteristics and medical institution characteristics) that influence treatment outcomes.

- To descriptively analyze the status of medical services provided by the registered Home medical centers and the demographic and clinical characteristics of the patients.

3. Names and Titles of Principal Investigator, Co-Investigators, and Person in Charge

1) Principal Investigator

| **Name** | **Affiliation** | **Title** | **Contact information** | **Role** |
| --- | --- | --- | --- | --- |
| Beom-seok Kim | Jungdong Korean Medicine Clinic | Director | 010-2772-1075  ds2jqu@hanmail.net | Overall Research Management, Participant Recruitment, Informed Consent Acquisition, Data Collection |
| Kwon-hee Kim | Geonganghanmaeul Korean Medicine Clinic | Director | 010-9932-1015  pine0426@daum.net | Participant Recruitment,  Informed Consent Acquisition,  Data Collection |
| Ho-yeol Bang | Dongbang Shintong Bubu Korean Medicine Clinic | Director | 010-3554-9750  rhrnal@daum.net | Participant Recruitment,  Informed Consent Acquisition,  Data Collection |
| Chang-hoon Kim | Haemalgeun Korean Medicine Clinic | Director | 010-4727-2533  lovehadela@naver.com | Participant Recruitment,  Informed Consent Acquisition,  Data Collection |
| Jeong-cheol Kim | Kim Jeong-cheol Korean Medicine Clinic | Director | 010-8954-7958  eegol@hanmail.net | Participant Recruitment,  Informed Consent Acquisition,  Data Collection |
| Hee-jun Sim | Seohwa Korean Medicine Clinic | Director | 010-7479-3393  tow10002@gmail.com | Participant Recruitment,  Informed Consent Acquisition,  Data Collection |

2) Co-Investigator

| **Name** | **Affiliation** | **Title** | **Contact information** | **Role** |
| --- | --- | --- | --- | --- |
| Dong-su Kim | College of Korean Medicine, Dongshin University | Assistant Professor | 010-6736-5661  dskim20@dsu.ac.kr | Research Planning, Research Leadership, Research Consultation. |
| Han-bit Jin | College of Korean Medicine, Dongshin University | Graduate Student | 010-9686-8655  hanbitjin22@gmail.com | Research Planning, Research Implementation |
| Hyeong-seon Jeon | College of Korean Medicine, Wonkwang University | Graduate Student | 010-5548-1423  hs14231423@naver.com | Research Planning, Research Implementation |
| Soo-min Song | Doctors of Korean Medicine for Health Rights | Director of Solidarity project | 010-4408-7719  s01044077719@gmail.com | Research Implementation |
| Ye-rin Bae | College of Korean Medicine, Dongshin University | Researcher | 010-4545-1240  dpfls1240@gmail.com | Research Implementation |
| Eun-ji Ahn | College of Korean Medicine, Dongshin University | Graduate Student | 010-9104-1515  ahneunji1015@daum.net | Research Implementation |

4. Name and Address of the Implementing Institution

1) Research Data Collection

| **Name of Medical Institution** | **Address** |
| --- | --- |
| Jungdong Korean Medicine Clinic | 2F, Wonheung Bldg., 181, Seokcheon-ro, Wonmi-gu, Bucheon-si, Gyeonggi-do |
| Geonganghanmaeul Korean Medicine Clinic | 3F, 168, Jangseungbaegi-ro, Wansan-gu, Jeonju-si, Jeollabuk-do |
| Dongbang Shintong Bubu  Korean Medicine Clinic | 1F, 111, Gohyeon-ro, Geoje-si, Gyeongsangnam-do |
| Haemalgeun Korean Medicine Clinic | 3F, 65, Buldang 21-ro, Seobuk-gu, Cheonan-si, Chungcheongnam-do |
| Kim Jeong-cheol Korean Medicine Clinic | 12-1, Cheongeun-ro, Jung-gu, Daejeon |
| Seohwa Korean Medicine Clinic | 4F, Haengsin Square, 126, Chungjang-ro, Deogyang-gu, Goyang-si, Gyeonggi-do |

| ※ Reasons for Selecting Home medical centers  1. What is a Home medical center?.  ‧ The Pilot Project for Long-term Care Home medical centers targets long-term care recipients of grades 1 to 4 who are assessed by a Korean Medicine doctor as needing home medical care due to mobility limitations.  ‧ Korean Medicine doctors, nurses, and social workers periodically visit the patient's home to provide visiting medical care, nursing, and linkage to community care services.  ‧ The Korean Medicine doctor provides visiting medical care at least once a month, the nurse provides visiting nursing at least twice a month, and the social worker provides periodic counseling to identify care needs and link services within the community. The specific services provided by the Korean Medicine doctor during the visiting medical care are as follows:  ① (Diagnosis) Examination including Mun-jin (inquiry/anamnesis), Mun-jin (auscultation/olfaction), Mang-jin (inspection), Chok-jin (palpation), Cheong-jin (auscultation), Ta-jin (percussion), An-jin (pressure diagnosis), and Maek-jin (pulse diagnosis).  ② (Prescription) Korean herbal medicine formulations listed in the 「Reimbursement List and Maximum Price Table for Korean Herbal Medicine Preparations」* **Examples: Single-herb extract preparations (e.g., Puerariae Radix extract powder, Glycyrrhizae Radix extract powder, Angelicae Gigantis Radix extract powder), complex-herb extract preparations (e.g., Gami-soyosan, Galgeun-tang, Gumi-ganghwal-tang).*  ③ (Disease Management) Management of main symptoms and concurrent diseases (e.g., acupuncture, moxibustion, cupping).  ④ (Examination) Korean Medicine examinations, such as temperament tests.  ⑤ (Referral) Referral to appropriate specialized medical institutions when necessary.  ⑥ (Patient Education and Counseling) Provision of patient status explanation and disease information, patient/caregiver education on health management, etc.  2. Reasons for Selecting Participating Institutions  ‧ The six Korean Medicine Home medical centers participating in this study are all affiliated with the 'Society for Korean Medicine Home Medical Care*'. These institutions possess a high level of understanding and practical experience in research based on Korean Medicine care and health care. They have previously served as essential collaborative partners in Korean Medicine care-related studies conducted by our research team. They were selected as institutions whose research capacity and willingness to participate have been verified.  *The Society for Korean Medicine Home Medical Care is a Practice-Based Research Network (PBRN) comprised of clinical practitioners and researchers dedicated to establishing a health care delivery model based on Korean Medicine. As of July 2025, it consists of 109 members. The society is actively expanding its research foundation through regular studies, external lectures, and practical sharing sessions. |
| --- |

2) Research Data Analysis

○ Department of Preventive Medicine, Dongshin University (Room 303, Daejeong Hall 4, 120-9, Dongsin Dae-gil, Naju-si, Jeollanam-do**)**

5. Research Supporting Institution

○ Korea Health Industry Development Institute (Health and Medical Administration Town, 187, Osongsaengmyeong 2-ro, Osong-eup, Heungdeok-gu, Cheongju-si, Chungcheongbuk-do)

6. Research Period

○ IRB Approval Date - 2028.12.31

7. Research participants

○ Patients from the six Home medical centers (designated as patient recruitment institutions for this study) who meet the Inclusion/Exclusion criteria.

- Long-term care Grade 1 is defined as "an individual requiring complete assistance from others in daily life due to physical and mental functional impairment," and Grade 2 is defined as "an individual requiring substantial assistance from others in daily life due to physical and mental functional impairment." The grade determination itself indicates a significant decline in the ability to perform daily living activities (ADL).

- Therefore, patients classified as long-term care Grade 3 or 4 will be included as study subjects, as those in the higher grades (1 and 2) are expected to show minimal change in condition.

| **Inclusion Criteria** | - Individuals who have received treatment at a Home medical center.  - Elderly individuals aged 65 years or older.  - Individuals classified as Long-term care Grade 3 or 4.  - Individuals who have clearly understood the study after receiving a thorough explanation, and who willingly decide to participate in the study and sign the consent form, either by themselves or through a legally authorized representative. |
| --- | --- |
| **Exclusion Criteria** | - Individuals who are expected to be unable to complete the 1-year follow-up (e.g., patients with a terminal illness having a life expectancy of less than 3 months, individuals planning to move to another region, etc.).  - Individuals who are currently participating in other clinical studies that may affect their participation in this study.  - Individuals whose condition is unstable due to an acute illness, and who are deemed to primarily require inpatient treatment rather than home medical care.  - Individuals with severe cognitive decline or communication disorders who are judged to be difficult to assess, even through themselves or a legally authorized representative. |

8. Expected Number of Study Participants and Rationale for Calculation

1) Target Enrollment

○ A total of 250 participants will be competitively recruited from the six research participant recruitment institutions over a period of one year starting from the first participant enrollment date. Following one year of follow-up observation, the final target is approximately 200 person-years.

2) Sampling Method

○ A registry study does not require the testing of predetermined statistical hypotheses, therefore, prior sample size calculation is unnecessary. However, the sample size will be determined within a realistically feasible range considering the research circumstances.

○ The number of study participants was calculated by referencing the figures from September 2024 to estimate the realistic potential recruitment.

- The six registry study participant recruitment institutions are distributed nationwide. As of September 2024, the total number of Long-term care Grade 3 and 4 patients across these participating institutions is approximately 370. The number of subjects was calculated assuming that the same patient population size will be maintained during the patient recruitment period of this study (2025–2026).

- Assuming that 70% of the 370 patients (i.e., 259 individuals) will participate in the study, and factoring in a 20% dropout rate, the final target is approximately 200 person-years. The 20% dropout rate was calculated to include all cases where sustained study participation may be difficult, such as complications, hospitalization or institutionalization due to condition deterioration, and death that may occur during the study period.

- Since Home medical centers provide services to patients through home visits, a high participation rate and a low dropout rate are expected.

| **Participating Institutions** | **Geonganghan**  **-maeul KMC** | **Jungdong KMC** | **Dongbang Shintong Bubu KMC** | **Haemalgeun KMC** | **Kim Jeong-cheol**  **KMC** | **Seohwa KMC**** | **Total number** |
| --- | --- | --- | --- | --- | --- | --- | --- |
| Number of Patients in September 2024 | 98 | 78 | 73 | 61 | 60 | - | 370 |
| Target Enrollment (70% of Total Patients) | 69 | 55 | 51 | 42 | 42 | - | 259 |
| Final Follow-up Target (Estimated 20% Dropout Rate) | 55 | 44 | 40 | 33 | 33 | - | 205 |

KMC is an acronym for Korean Medicine Clinic.

* This institution was newly selected in 2025, therefore the number of eligible subjects cannot be calculated.

○ Additionally, this study aims to identify treatment responders by performing a statistical analysis of factors influencing treatment effects, and the number of study subjects was calculated based on this.

- A multivariate logistic regression analysis is planned for K-ADL, which is a continuous variable. Ten covariates influencing treatment success were selected in advance.

- The covariates are items from the Home medical center Comprehensive Assessment Record, specifically: Sex, Age, Presence of Cardiovascular Disease, Presence of Diabetes, Presence of Hypertension, Presence of Hyperlipidemia, Pre-treatment K-FRAIL score, Pre-treatment Five-times Chair and Stand Test (5CST) score, Pre-treatment Timed Up and Go Test (TUG test) score, and Pre-treatment 10-stair climbing ability score.

- Since generally 20 subjects are required per variable, 10 variables require $10 \times 20 = 200 study subjects.

9. Recruitment of Study Participants

○ Study Participant Recruitment Methods

- This study is a patient registry study, and the subject recruitment process is part of the general clinical practice.

- Since medical care at the Home medical center is provided through home visits, the director of the participating institution, acting as a co-investigator, will visit the subject's home to thoroughly explain the IRB-approved research explanation document and consent form to the subject or their legally authorized representative before recruitment.

- Additionally, the recruitment advertisement for this study will be posted on the bulletin board within the study participant recruitment institutions and displayed as a pop-up on their websites

- Recruitment will be conducted for one year following the IRB approval date, and the entire recruitment process will be overseen by co-investigator Dr. Kim, Dongsoo.

10. Informed Consent of Study Participants

1) Consent Form

○ Two types of explanation documents and consent forms approved by the Public Institutional Review Board (IRB) will be used.

- Study Participation Consent Form: A form seeking consent solely for participation in this patient registry study.

- Consent Form for Third-Party Provision of Personal Information and Secondary Research Use: A separate form seeking consent for providing the participant's information collected through the study to the National Institute for Korean Medicine Development(NIKOM) and for secondary use in future research.

○ The investigator must clearly explain that participants may participate in this patient registry study even if they only consent to the 'Study Participation Consent Form' and do not consent to the 'Consent Form for Third-Party Provision of Personal Information and Secondary Research Use'.

- In this case, the participant's information will only be used for this study and will not be provided to the National Institute for Korean Medicine Development.

○ The consent of the participants will be obtained by the research staff belonging to the participating institutions, who will directly explain the study to the participants and receive their written consent.

- The consent process will take place in the participant's home, ensuring a stable and quiet environment that allows for sufficient and smooth communication between the research staff and the participant.

- During this process, personal identification information, such as the participant's name, must be managed securely to prevent access by anyone other than the research staff.

- The research staff must fully explain the following to the participants:

• The purpose and background of the study

• The study procedures and estimated time commitment

• The details of the examinations and data to be collected

• Measures for personal information protection and confidentiality

• The fact that the collected data may be transferred to the co-investigational institution for analysis

• That participation in the study is a voluntary decision, and they can withdraw at any time without penalty

- Should the participant have any questions, the research staff must take sufficient time to explain and respond sincerely.

- If the participant fully understands the explanation and voluntarily expresses their intent to participate, the research staff will have them complete and sign the written consent form on the same day before including them in the study, ensuring the decision is made autonomously without coercion or inducement.

- This written consent signifies agreement to participate for the entire duration of the study; however, an additional re-consent form may be obtained during the study period depending on the participant's request or a change in their capacity to consent, and if necessary, the re-consent procedure must be conducted accordingly.

2) Assessment of Participant's Capacity to Consent

○ Plan for Assessing Participant's Capacity to Consent

- The capacity to consent will be comprehensively determined based on the following: 1) whether the participant understands the study-related information, 2) whether the participant can logically handle the information about the study, and 3) whether the participant has the ability to clearly express their choice regarding whether or not they wish to participate in the study.

- If, based on the above assessment of capacity to consent, the participant is judged to have sufficient capacity, their own consent will be obtained.

- If, based on the above assessment, consent for study participation is deemed difficult, the participant will be classified as a vulnerable participant, and written assent from the participant and consent from the legally authorized representative (LAR) will be obtained.

- Assessment of the LAR's capacity to consent, if required, will be conducted through examination by the Korean medicine doctor belonging to the participating institution.

- If the LAR's capacity to consent is diminished, consent will be obtained from another LAR, or if there is no LAR with the capacity to consent, the patient will not be enrolled.

- The LAR's consent must be completed by the LAR. In this case, documents such as a family relationship certificate (or equivalent) to prove the LAR's consent will be collected and stored, as they are essential materials to objectively prove that consent was obtained from the lawful representative for the protection of vulnerable participants' rights.

• Personal information of a third party, such as a family relationship certificate, will be immediately and completely destroyed using a document shredder after confirming the identity of the LAR.

○ Procedures for Minimizing Risks to Participants

- The director of the participating institution will reassess the participant's capacity to consent periodically if, during any home visit, they determine there has been a significant change in the participant's cognitive function or communication ability.

• Reasons for obtaining re-consent include: if a participant initially entered the study with their own consent but their capacity to consent decreases during follow-up, necessitating consent from a legally authorized representative (LAR); or if the participant or LAR requests a re-explanation of the consent information.

- In such cases, consent will be obtained from the LAR using the same procedure as the initial consent acquisition.

- If the LAR's capacity to consent is diminished, efforts will be made to obtain consent from an alternative LAR who has the capacity to consent. If no LAR with the required capacity is available, the patient will be withdrawn from the study.

- Participants will be repeatedly informed that they may decide to continue or stop the study at any time, and that there will be no penalty for discontinuing participation.

11. Research Methods

1) Study Design

○ This study is a registry-based observational study that aims to continuously and systematically collect data in a standardized format from patients exposed to specific factors, in order to evaluate long-term health outcomes and the effectiveness of service provision.

- Registry studies are generally conducted to identify and improve the characteristics of a specific disease, the clinical effectiveness of treatment, and the quality of services. They have the advantage of being adaptable according to the researchers' objectives, without the fixed framework of a randomized controlled trial.

- Various forms exist, such as disease registries and health services registries, and they can be operated by combining multiple objectives rather than a single purpose.

- This study aims to register patients who have received services from the Korean Medicine Home medical center. As assessments for frailty and chronic pain will be performed among the registered patients, the registry will be operated by combining the objectives of a health services registry and a disease registry.

○ In this study, exposure to the Korean medicine Home medical center service is defined based on the time of the comprehensive assessment record, and a mixed (retrospective + prospective) data collection structure will be applied as follows:

- The Home medical center assesses the initial status of a patient during the first consultation using the 'Comprehensive Assessment and Care Plan Record Sheet for Long-term Care Home medical centers,' which is Form No. 5 of the Pilot Project Guideline for Long-term Care Home medical centers.

- Therefore, a record of the first comprehensive assessment remains for all patients, and that time point is defined as the time of exposure.

- For new Home medical center patients, all measurement variables, including the comprehensive assessment record sheet, will be collected prospectively.

- For existing Home medical center patients, the time of the first comprehensive assessment is considered the exposure to the Home medical center treatment. The past comprehensive assessment record sheets will be collected retrospectively, while the measurement variables for the study and the comprehensive assessment record sheets during the study period will be collected prospectively.

2) Definition of Functional Decline, Frailty, and Chronic Pain

○ Definition of Functional Decline

- Function refers to an individual's abilities in body functions, activity performance, and social participation. Functional decline is defined as a state in which these abilities are impaired or restricted.

- According to the Long-Term Care Eligibility Assessment Tool (Janggi-yoyang Injeong Josapyo), the applicant's ability to perform daily activities and the degree of functional decline are comprehensively assessed across five domains—physical function (12 items), cognitive function (7 items), behavioral change (14 items), nursing procedures (9 items), and rehabilitation (10 items)—and utilized for long-term care grade determination.

- Since this grade determination reflects physical and cognitive functional decline, individuals with a long-term care grade can be defined as functionally impaired older adults.

- In this study, the level of functional decline change among participants will be measured using the K-ADL (7 points: completely independent, 21 points: completely dependent).

○ Frailty

- Frailty is defined as a state of vulnerability resulting from an overall decline in function due to aging, which leads to reduced resilience and physiological reserve capacity, making the body unable to respond appropriately to external stimuli. It is a state where the risk of disease, functional disability, dependence, and death increases.

- In this study, changes in the participant's level of frailty will be assessed using the K-FRAIL. A score of 0 indicates normal, 1-2 indicates pre-frailty, and 3-5 indicates frailty.

○ Chronic Pain

- The WHO, in ICD-11, defines chronic pain as "pain that persists or recurs for more than 3 months, and is a disease state that can reduce quality of life, regardless of whether there is a clear cause."

- Chronic pain is pain that lasts for a certain period or does not disappear even after the injury has healed, and it is considered an independent disease rather than just a symptom.

- Chronic pain increases the persistent use of analgesics; reports indicate that approximately 75% of chronic pain patients have been prescribed at least one analgesic.

- In this study, chronic pain will follow the definition provided by ICD-11 and will be assessed using the Numerical Rating Scale (NRS).

3) Participant Enrollment

○ An identification code is assigned and recorded according to the institution obtaining consent and the order of enrollment, and the participant's name is indicated using the first three letters as initials.

- The code is indicated in the order of: Implementing Institution Code - Implementing Year - Enrollment Order.

- The notation example is as follows: JDC-2026-015: The 15th patient enrolled in the study in 2026 at Jungdong Korean Medicine Clinic.

| **Name of Institution** | **Code** |
| --- | --- |
| Jungdong Korean Medicine Clinic | JDC |
| Geonganghanmaeul Korean Medicine Clinic | GGH |
| Dongbang Shintong Bubu Korean Medicine Clinic | DBS |
| Haemalgeun Korean Medicine Clinic | HME |
| Kim Jeong-cheol Korean Medicine Clinic | KJC |
| Seohwa Korean Medicine Clinic | SHC |

| **Participant identification code** | **Participant initial** |
| --- | --- |
| 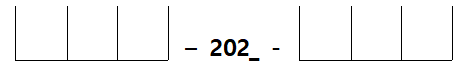 | \|  \|  \|  \| \| --- \| --- \| --- \| |
| □ NA (Screening Fail) |  |

**4) Research Process**

| **Screening** | Informed Consent for Study Participation | - Obtaining Consent from the Participant and Legally Authorized Representative (LAR) |
| --- | --- | --- |
|  | Screening Assessment | - Confirmation of Inclusion/Exclusion Criteria  - Assignment of Participant Number  - Personal Information (Name, Date of Birth, Sex, Address) |
| **Measure 1** | Baseline Assessment | - K-Activities of Daily Living (K-ADL)  - K-FRAIL (Fraility)  - Numerical Rating Scale (NRS, pain)  - Medication History |
| **Measure 2** | Follow-up Assessment  at 1 Year | - K-Activities of Daily Living (K-ADL)  - K-FRAIL (Fraility)  - Numerical Rating Scale(NRS, pain)  - Medication History  - Client Satisfaction Questionnaire (CSQ) |
| **Continuous Measurement** | Follow-up over 1 Year | - Adverse Events  - Visiting Check-up and Work Records (Physician, Nurse, Social Worker) of the Long-term Care Home medical center  - Numerical Rating Scale(NRS, pain) |
| **Comprehensive Assessment** | Regular Assessment | - Comprehensive Assessment and Care Plan Record of the Long-term Care Home medical center |

| **Measure** | **Before**  **the study** | **Screening*** | **Measure 1** | **Measure 2** | |
| --- | --- | --- | --- | --- | --- |
| **Months** |  | **0** | **0**  **(+30days)** | **12**  **(+-30days)** | |
| **Obtaining Informed Consent** |  | ● |  |  | |
| **Confirmation of Inclusion/Exclusion Criteria** |  | ● |  |  | |
| **Assignment of Subject Number** |  | ● |  |  | |
| **Personal Information (Name, Date of Birth, Sex, Address)** |  | ● |  |  | |
| **Comprehensive Assessment Record**** | 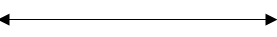 | | | | |
| **K-ADL K-FRAIL, NRS, Medication History** |  |  | ● | ● | |
| **Client Satisfaction Questionnaire** |  |  |  | ● | |
| **Adverse Events, Visiting Check-up and Work Records (Physician, Nurse, Social Worker), NRS** |  |  | 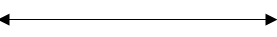 | | |
| * If the screening meets the Inclusion Criteria, the Initial Measurement can be performed.  ** Since measurements are taken annually for each participant, data should be collected according to the timing of the medical institution's measurement schedule. Existing patients of the Home medical center will have retrospective data collected, while new patients will only have prospective data collected. | | | | |  |

○ The schedule for the participants is as follows:

- When the willingness to participate in the study is confirmed, Obtaining Consent, Confirmation of Inclusion/Exclusion Criteria, Assignment of Participant Number, and Personal Information (Name, Date of Birth, Sex, Address) are verified.

- Baseline Assessment (K-ADL, K-FRAIL, NRS, Medication History) is conducted to evaluate the patient's status.

- Participants receive services from the Home medical center for one year, and at every visit, Visiting Check-up and Work Records (Physician, Nurse, Social Worker), NRS, and Adverse Events are recorded.

• This study is an observational study, and there are no additional medical visits required due to participation in the study. The frequency of visits during the study period will follow the Home medical center's Care Plan established under the judgment of the assigned medical team according to each participant's condition.

• However, as a minimum standard for visits, a Korean medicine physician will visit at least once a month, and a nurse will visit at least twice a month, in accordance with the pilot project guidelines for the Long-term Care Home medical center.

- After one year of treatment, a Follow-up Assessment (K-ADL, K-FRAIL, NRS, Medication History) and satisfaction evaluation are conducted to evaluate the patient's status.

• Since Activities of Daily Living (K-ADL) or frailty (K-FRAIL) change gradually due to their nature, a minimum follow-up period of one year is set to observe clinically significant changes.

- The Comprehensive Assessment Record will be measured according to the process of the participating research institution, irrespective of the schedule for the Baseline Assessment and Follow-up Assessment.

- Treatment at the Home medical center will continue even after the study is completed.

○ The time required for the study is as follows:

- During the study period, participants must receive a Baseline Assessment, a Follow-up Assessment, and at least one visiting medical consultation, with each visiting medical consultation taking approximately 30 minutes.

- The Baseline Assessment and Follow-up Assessment will take approximately 1 hour.

○ The study completion is as follows:

- Study participation is concluded when the participant completes the final Follow-up Assessment at the 1-year mark after enrollment.

12. Observation Items

1) Observation Items

○ Personal Information (Name, Date of Birth, Sex, Address)

- For the purpose of secondary data linkage, this study must collect Name, Date of Birth, Sex, and Address as linking keys. These items are mandatorily collected during the clinical care process and can be secured regardless of participation in the study.

- The collected personal information can only be accessed by the Principal Investigator and Co-Investigators. The data will be stored in a cabinet with a locking device and on password-protected computers, and measures will be taken to ensure that only authorized personnel can access it.

○ Comprehensive Assessment Record, Visiting Check-up and Work Records (Physician, Nurse, Social Worker) (Attachment 1-4)

- The following forms are used: Form No. 5, "Long-term Care Home medical center Comprehensive Assessment and Care Plan Record," and Forms No. 6, No. 7, and No. 8, "Long-term Care Home medical center Visiting Check-up Record (Physician)," "Long-term Care Home medical center Visiting Check-up Record (Nurse)," and "Long-term Care Home medical center Work Record (Social Worker)" from the guidelines of the Long-term Care Home medical center Pilot Project.

- The Comprehensive Assessment Record is completed once a year, and the Visiting Check-up and Work Records are recorded at every visiting medical consultation.

- The long-term care recognition number, validity period, and guardian information within the Comprehensive Assessment Record are not collected, and the long-term care recognition number in the Visiting Check-up and Work Records is also deleted and not collected.

- The utilization plan for the Comprehensive Assessment Record, Visiting Check-up, and Work Records is as follows.

• Analysis of Participant Characteristics: The Comprehensive Assessment Record will be utilized to determine the diverse baseline characteristics of the participants at the study initiation, including demographic information, comorbidities, physical and cognitive function, and medication history. This data will be used as the basis for future analysis of the effectiveness of the home medical care service.

• Understanding Medical Service Content: The Visiting Check-up and Work Records will be used to quantitatively analyze the specific service content provided during the study period, such as the type, frequency, duration, and details of treatments.

| ※ Information Collected via Comprehensive Assessment Record and Visiting Check-up Records  ‧ Comprehensive Assessment Record: Date of Visit, Institutional Information, Participant Information (Name, Date of Birth, Sex, Long-Term Care Grade, Address), Health/Disease Status (Primary problems requested to be resolved, Comorbidities under treatment, Medication History, etc.), Physical Status (Gait ability and muscle strength test, Nutritional status and eating behavior, etc.), Mental Status (Cognitive function, Depression, etc.), Possibility of Independent Living (Level of consciousness, Physical function, Cognitive function, etc.), Items requiring Medical Procedures and Health Management, Social/Environmental Assessment (Need for assistance with daily life, Economic status, Social relations, etc.), Service Utilization Status (Medical services, Long-term care services, etc.)  ‧ Visiting Check-up Record (Physician): Institutional Information, Participant Information (Name, Date of Birth, Sex, Long-Term Care Grade, Address), Service Provision Time, Reason for Visit, Independence in Physical Function, Independence in Cognitive Function, Examination and Consultation Content, Invasive Procedure Content, Nursing Instructions, Future Plan  ‧ Visiting Check-up Record (Nurse): Institutional Information, Participant Information (Name, Date of Birth, Sex, Long-Term Care Grade, Address), Reason for Visit, Service Provision Time, Accompanying Person, Visit Content (including Medication Management), Health Status, Blood Pressure/Pulse, Body Temperature/Blood Sugar, Weight Change, Delirium, Falls, Urinary/Fecal Incontinence, Future Plan  ‧ Visiting Check-up Record (Social Worker): Institutional Information, Participant Information (Name, Date of Birth, Sex, Long-Term Care Grade, Address), Consultation Content, Community Linkage |
| --- |

○ K-ADL (Attachment 5)

- It is a Korean version of the Activities of Daily Living measurement tool, developed in 2002 by the Functional Assessment Research Group of the Korean Geriatrics Society, commissioned by the Ministry of Health and Welfare, to suit the Korean culture.

- It consists of seven questions. A higher score indicates that the individual performs basic daily activities well independently, while a score closer to 0 indicates a greater need for assistance from others.

○ K-FRAIL (Attachment 6)

- This tool is widely used for the diagnosis of frailty and is suitable for application in primary care settings.

- It consists of five frailty criteria and classifies individuals into three frailty stages based on the total score: Not Frail (score 0), Pre-frail (score 1-2), and Frail (score 3-5).

○ NRS (Attachment 7)

- The Numeric Rating Scale (NRS) is a tool that digitizes the Visual Analogue Scale (VAS), expressed by numbers from 0 to 10, where 0 is assumed to be 'no pain at all' and 10 is assumed to be 'the worst pain imaginable,' asking the patient to state the current level of pain numerically.

- Research results indicate that NRS is preferred over VAS by patients with chronic pain because it is easier to understand and perform.

○ CSQ (Attachment 8)

- The questionnaire used in the patient satisfaction study on home medical care is utilized.

- The Client Satisfaction Questionnaire (CSQ) is known through prior research to have high internal consistency and a strong correlation with patient symptom reduction and continued treatment rates.

2) Measurement Methods

○ Questionnaires

- K-FRAIL, NRS, K-ADL, Comprehensive Assessment Record, and Visiting Check-up Records (Physician, Nurse, Social Worker) will be administered by research personnel who have been trained in advance.

- These assessment items will be conducted during visiting medical consultation in a prepared space with a stable and quiet environment to ensure smooth communication between the assessor and the participant. The assessor will complete the forms based on direct observation or interview for each item.

- The Visiting Check-up Records will be completed directly by the respective professional staff (physician, nurse, social worker) during the visiting medical consultation.

- If the respondent is unable to self-report due to issues such as language, hearing, or cognitive function, proxy responses by a legal representative or care provider are permitted. This will be determined by the researcher based on a prior assessment of the specific case.

• Proxy responses for the National Approved Statistics (Survey of Persons with Disabilities) are permitted for: 1. Parents, 2. Spouses, 3. Siblings, 4. Children, 5. Neighbors or Relatives, 6. Activity Assistants, Caregivers, etc., 7. Institutional workers.

• This study will apply the stricter criteria of a preceding study (Cho Mi-hee, 2024) to accurately understand the status of the participants.

• Proxy respondents in this study are limited to a legal representative or care provider. A care provider is defined as someone who performs substantial care for an average of 3 hours or more per day, and includes the following categories: (1) Family caregiver, (2) Certified caregiver, (3) Activity assistant for the disabled.

○ Satisfaction Questionnaire

- The Client Satisfaction Questionnaire (CSQ) will be conducted exclusively by Investigator Song Su-min, who is affiliated with an entity independent of the participating research institution, to ensure the acquisition of objective evaluations.

- The survey targets the patient themselves who participated in both the Baseline Assessment and the Follow-up Assessment during the study period and received visiting medical consultation at least once.

- If self-reporting is difficult, this survey does not permit proxy responses, and the assessment at that specific time point will be excluded.

- The survey principle is an in-person, face-to-face interview. If a face-to-face interview is impossible, it may be replaced by a telephone interview. Even in the case of a telephone interview, the same survey guidelines and questions are applied, and the interview is conducted only after thoroughly verifying the respondent's identity.

3) Method of Collection and Management

○ Evaluator Training Method

- To ensure consistency across all evaluations, a Standard Operating Procedure (SOP) will be created, and a workshop will be conducted for all evaluators before the start of the study.

- Early in the study, inter-rater reliability will be verified by having two evaluators assess the same few patients to see how consistent their results are. If the results are low, re-education will be implemented.

○ Data Collection Methods

- The data collection methods are finalized through consultation with the research team and the staff in charge of investigating medical records, surveys, and electronic data.

- The Case Report Forms (CRFs) will be completed in paper form. The original copies will be stored at the participating research institution, and copies will be sent to Investigator Bae Ye-rin at Dongshin University.

- The original copies of the Comprehensive Assessment Record and the Visiting Check-up Records (Physician, Nurse, Social Worker) will be stored at the participating research institution. Copies will be sent to Investigator Bae Ye-rin at Dongshin University after deleting personal information other than the participant’s personal information (Name, Date of Birth, Sex, Address) scheduled for collection in the CRFs.

- The transferred data will undergo a cleaning process and will be systematically managed in the electronic Case Report Form (eCRF; iClick) provided by the Korean Medicine Innovative Technology Development Project Group to ensure research transparency.

13. Criteria and Methods for Evaluating Effectiveness

1) Characteristics of the Home medical center and Patients

○ The medical consultation information of the Home medical center and the general characteristics of the patients included in the Comprehensive Assessment Record and the Visiting Check-up and Work Records will be analyzed.

- The baseline characteristics of patients at the Korean medicine Home medical center will be analyzed descriptively.

- The results of this analysis will be utilized as supporting evidence for the interpretation of future effectiveness.

2) Primary outcome

○ K-ADL

- The K-ADL score, which indicates the patient's functional status, has been set as the primary evaluation indicator.

- The change in the K-ADL score will be comparatively evaluated between the pre-treatment (Baseline Assessment) and post-treatment (Follow-up Assessment).

3) Secondary outcome

○ K-FRAIL and NRS will be evaluated using the change in score.

○ The Client Satisfaction Questionnaire (CSQ) assessment scores will be evaluated descriptively after registry participation.

○ Comprehensive Assessment Record, Visiting Check-up and Work Records

- Given the nature of registry-based analysis, rather than rigidly setting specific statistical hypotheses in advance, the plan is to gradually expand the scope and methods of analysis according to the questions that arise during the study period.

- The items in the Comprehensive Assessment Record are categorized into score variables and event variables based on the characteristics of the variables. Accordingly, item-by-item analysis will be performed based on the criteria for effectiveness evaluation presented in this study.

14. Anticipated Side Effects, Precautions, and Management

1) Additional Side Effects Due to Participation in Observational Study

○ No additional side effects are anticipated to occur due to study participation.

- This study is an observational study conducted within the routine clinical care environment of a Home medical center, and it does not include separate invasive examinations or additional treatments for research purposes.

- Considering these study characteristics, this study will only collect Serious Adverse Events (SAEs), which are defined as follows:

• Death

• A situation where home-based treatment is impossible due to the necessity of long-term hospitalization or institutionalization exceeding 30 days.

2) Actions and Reporting Upon Occurrence of Adverse Events

○ The research team will confirm the occurrence of Serious Adverse Events (SAEs) through visiting medical consultation or contact from the participant or guardian. Upon awareness of an occurrence, prompt action and reporting will be carried out according to the following procedures:

- In Case of Necessity for Hospitalization or Institutionalization

• Status Evaluation and Recommendation: The Korean medicine doctor at the participating research institution will comprehensively assess the patient's status during routine visiting medical consultation, including vital signs, subjective symptoms, and clinical observation and evaluation. If the assessment determines that intensive treatment or care exceeding the scope of home medical care is necessary, hospitalization or institutionalization will be strongly recommended to the participant and their guardian.

• Medical Institution Linkage: Once hospitalization or institutionalization is decided, the Korean medicine doctor will provide information about an appropriate higher-level medical institution or nursing facility for the patient's condition and assist in ensuring smooth linkage, such as issuing a referral if necessary.

• Study Participation Discontinuation Processing: At the point where long-term hospitalization or institutionalization exceeding 30 days makes further follow-up observation in this study impossible, the participant will be processed as discontinued from study participation, and the reason and progress will be documented in detail in the Case Report Form (CRF).

- In Case of Death During Home medical care

• Awareness and Action: Immediately upon confirming the participant's death, whether through contact from the guardian or direct observation by the Korean medicine physician, the Principal Investigator (PI) will take prompt action according to the Clinical Practice Guidelines for End-of-Life Care and related regulations.

• Issuance of Death Certificate: If necessary, the Korean medicine physician will issue a death certificate in accordance with relevant regulations and offer condolences and guidance on necessary administrative procedures to the bereaved family.

• Recording and Study Processing: The fact, time, and circumstances of death will be verified and officially recorded in the medical record. At the point when death makes further follow-up observation in this study impossible, the participant will be processed as discontinued from study participation and documented in detail in the CRF.

- Reporting to the Institutional Review Board (IRB)

• Although no Serious Adverse Events resulting from study participation are anticipated, due to the study targeting the elderly, the occurrence of death, hospitalization, and institutionalization is expected during the study period.

• Therefore, at the time of continuing review by each institution, the cumulative number of deaths, hospitalizations, and institutionalizations across all participating institutions will be collected and reported together.

15. Data Analysis and Statistical Methods

1) Characteristics of the Home medical center and Patients

○ Prior to analysis, descriptive statistics will be calculated cross-sectionally for the patient and Home medical center characteristics.

- Continuous variables will be presented as mean $\pm$ standard deviation or median (interquartile range). Categorical variables will be presented as frequency (%).

○ To identify factors influencing the intervention effect, a multivariate regression model will be utilized, including key patient and institutional characteristic variables as independent variables.

- A model will be constructed that includes interaction terms with individual characteristics, in addition to the time-by-group interaction, to evaluate whether the intervention effect differs according to specific factors.

2) Primary outcome: K-ADL

○ This study is a single-arm observational study without a control group, and the scores will be compared before and after the study period.

- A Paired t-test (or Wilcoxon signed-rank test) will be performed.

- The statistical significance level will be set at less than 0.05.

| Hypothesis Setting:  Null Hypothesis (H₀): The K-ADL score at the Follow-up Assessment is the same, with no change compared to the Baseline Assessment.  Alternative Hypothesis (H₁): The K-ADL score at the Follow-up Assessment is not the same compared to the Baseline Assessment. |
| --- |

3) Secondary outcome

○ K-FRAIL: Paired t-test (or Wilcoxon signed-rank test) will be performed.

○ NRS: Repeated Measures ANOVA (or Friedman Test) will be performed.

○ CSQ: Since it is measured only at the end of the study, descriptive statistics will be performed.

○ The statistical significance level will be set at less than 0.05

4) Comprehensive Assessment Record, Visiting Check-up and Work Records

○ The data from the Comprehensive Assessment Record and the Visiting Check-up and Work Records consist of continuous and event variables across various domains, and appropriate statistical analysis methods will be applied according to the properties of each variable.

- Different methods will be applied to repeated measures data used to analyze changes over time, depending on the number of measurement points.

- If there are two measurement points, changes in continuous variables will be analyzed using a Paired t-test, and categorical variables will be analyzed using McNemar’s test.

- If there are three or more measurement points, continuous variables will be analyzed using Repeated Measures ANOVA or a Linear Mixed Model, and categorical variables will be analyzed using Cochran’s Q test or Generalized Estimating Equations (GEE).

- The significance level for all statistical analyses will be set at less than 0.05

5) Analysis of Treatment Responders

○ A case where the change in the pre- and post-treatment K-ADL score is equal to or greater than the pre-defined Minimum Clinically Important Difference (MCID) value will be defined as the 'Clinically Significant Improvement Group', and predictive factors influencing this group will be explored.

- Multivariate logistic regression analysis will be performed, adjusting for factors such as sex, age, presence of cardiovascular disease, presence of diabetes, presence of hypertension, presence of hyperlipidemia, pre-treatment K-FRAIL score, pre-treatment Five-times chair and stand test (5CST) score, pre-treatment Timed Up and Go test (TUG test) score, and pre-treatment 10-stair climbing ability score.

6) Missing Data Management

○ The missing data anticipated in this study will be managed by applying Multiple Imputation (MI) to minimize bias in statistical analysis and ensure the reliability of the results.

- Imputation Method: Missing values will be predicted using a multivariate imputation algorithm based on chained equations, creating a total of 20 plausible complete datasets.

- Imputation Model Variables: To enhance the accuracy of the imputation model, both the primary outcome variable (K-ADL) and the key predictor variables (age, sex, etc.) will be included.

- Analysis and Pooling: The primary analysis will be independently performed on each of the 20 generated datasets, and the results will then be pooled into a single final estimate according to Rubin's rules.

16. Withdrawal of Consent and Dropout

1) Criteria for Dropout

○ The research participant may drop out of the study if they fall under any of the following cases during the course of the study:

- The participant voluntarily withdraws consent to participate during the study.

- The participant or their legal representative loses the capacity to consent during the study period.

- It is confirmed that the participant does not meet the inclusion criteria or meets the exclusion criteria.

- A Serious Adverse Event (SAE) occurs.

- Other cases where the Principal Investigator or person in charge determines that continuing the study is not appropriate for the participant.

○ Data collected up to the point of dropout will be included in the study. However, if the participant explicitly withdraws consent for the use of their personal information and data at the time of signing the consent form, during the study, or even after dropout, the corresponding research data will be excluded from the analysis.

○ Participants who drop out of this study will not be re-enrolled in the study thereafter.

2) Criteria for Study Termination

○ The study may be terminated prematurely if it is determined that the research poses a significant risk to the safety and well-being of the participants.

3) Withdrawal of Consent

○ Even though the participant voluntarily consented to participate in this study, they have the right to withdraw consent at any time during the research and will not face any disadvantage as a result of the withdrawal.

17. Risks and Benefits to Participants

1) Anticipated Risks

○ This study is based on the standard home medical care service and evaluation questionnaires provided within the existing clinical setting, and no additional procedures or invasive interventions are performed due to study participation; therefore, there are no anticipated risks.

2) Anticipated Benefits

○ The following direct or indirect benefits may be provided to the participants:

- Continuous confirmation and management of the patient's health status are achieved through regular comprehensive assessment and status monitoring.

- There is a possibility of improvement in various areas, such as Activities of Daily Living (ADL) and pain, following the intervention.

- By utilizing their clinical information as evidence for improving future care policies, participants can indirectly contribute to the advancement of public health and medical care.

18. Compensation for Study Participation

○ As compensation for study participation, hygiene products and Korean medicine patches worth 20,000 KRW will be provided once.

19. Measures for Participant Safety and Personal Information Protection

1) Information Collection for Routine Medical Care and Patient Management

○ This study will collect the following information for the purpose of the participant's medical care and assessment:

- Personal Information: Name, Sex, Date of Birth, Address

- Sensitive Information: Patient characteristic data within the Comprehensive Assessment Record and Visiting Check-up Records, and results from various questionnaires.

- No participant information from the Comprehensive Assessment Record other than the collected personal information will be gathered.

| ※ Information Collected via Comprehensive Assessment Record and Visiting Check-up Records  ‧ Comprehensive Assessment Record: Date of visit, Institutional information, Participant information (Name, Date of Birth, Sex, Long-Term Care Insurance Grade, Address), Health/Disease status (Key problems requiring resolution, Comorbid diseases under treatment, Medication details, etc.), Physical status (Walking ability and muscle strength test, Nutritional status and eating behavior, etc.), Mental status (Cognitive function, Depression, etc.), Potential for independent living (Level of consciousness, Physical function, Cognitive function, etc.), Medical treatment and health management required items, Social/Environmental assessment (Need for assistance in daily life, Economic situation, Social relationships, etc.), Service utilization status (Medical services, Long-Term Care Services, etc.)  ‧ Visiting Check-up Records (Physician): Institutional information, Participant information (Name, Date of Birth, Sex, Long-Term Care Insurance Grade, Address), Service provision time, Reason for visit, Autonomy in physical function, Autonomy in cognitive function, Examination and consultation details, Details of invasive procedures, Nursing instructions, Future plan  ‧ Visiting Check-up Records (Nurse): Institutional information, Participant information (Name, Date of Birth, Sex, Long-Term Care Insurance Grade, Address), Reason for visit, Service provision time, Companion, Details of visit (including medication management), Health status, Blood pressure/Pulse, Body temperature/Blood glucose, Weight change, Delirium, Falls, Urinary/Fecal incontinence, Future plan  ‧ Visiting Check-up Records (Social Worker): Institutional information, Participant information (Name, Date of Birth, Sex, Long-Term Care Insurance Grade, Address), Consultation details, Community linkage |
| --- |

○ All this information consists of items routinely collected during the process of routine medical care and patient management, and the scope of data collection for this study does not exceed this.

○ Separate from the 'Informed Consent Form for Study Participation', the 'Consent Form for Third-Party Provision and Secondary Research Use of Personal Information' will be provided to the participants.

- Only if the participant signs this separate consent form will the collected research information be provided to a third party and used for secondary research purposes.

- Participants may still participate in this patient registry study simply by consenting to the 'Informed Consent Form for Study Participation,' even if they do not consent to the 'Consent Form for Third-Party Provision and Secondary Research Use of Personal Information.'

- If Consent is given for the 'Consent Form for Third-Party Provision and Secondary Research Use of Personal Information': The participant's information will be provided to the National Institute for Korean Medicine Development (user-defined translation) and may be linked and combined with databases from national public institutions (Health Insurance Review and Assessment Service, Statistics Korea, National Health Insurance Service, Korea Disease Control and Prevention Agency, National Cancer Center, etc.) for use in other research.

- If Consent is NOT given for the 'Consent Form for Third-Party Provision and Secondary Research Use of Personal Information': The participant's information will not be provided to the National Institute for Korean Medicine Development and will be used solely for the purpose of this study.

2) Confidentiality of Personal Information

○ Records that could identify the participant will be guaranteed as confidential, and the participant's identity will be maintained confidential even when the results of the observational study are published.

- All research data containing collected patient personal information will only be accessible to the Principal Investigator and co-investigators.

- The researcher must understand that by signing the contract for this observational study, the sponsor or monitor may review or copy the participant's chart and Case Report Form (CRF) for verification purposes.

3) Storage Method and Location of Research Data

○ Related data will be stored in a location where access by unauthorized persons is prevented by installing a locking device (lock) to ensure that only authorized personnel can access it.

- Data will be stored in locked cabinets in the research team's consultation rooms and on password-restricted computers in the offices of the analysts and evaluators, ensuring access is limited to the Principal Investigator and co-investigators only.

4) Data Retention Period

○ In accordance with Article 15 of the Enforcement Rule of the Bioethics and Safety Act, research data (Institutional Review Board (IRB) review results, consent forms, personal information collection/use/provision status, and final study report) will be retained for a minimum of three years after the completion of the study. Data provided to analysts and evaluators will also be retained for a minimum of three years from the time the study is completed.

5) Data Destruction Method

○ All research data, including electronic documents (protocol, IRB review results, final study report, etc.), will be permanently destroyed after the retention period using a document shredder or by deleting the electronic files.

○ Information provided to the Korean Medicine Innovative Technology Development Project Group via iClick will be retained for up to 10 years after study completion and then deleted.

20. References

- Statistics Korea. (2023). Future Population Projections. Statistics Korea. <https://kostat.go.kr/>
- Republic of Korea Policy Briefing. (2018, November 19). Announcement of Community Care Basic Plan (Phase 1: Senior Community Care) [Press Release]. Ministry of Health and Welfare.
- Republic of Korea Policy Briefing. (2021, November 17). Integrated Community Care. <https://www.korea.kr/special/policyCurationView.do?newsId=148866645#L1>
- Enforcement Decree of the Long-Term Care Insurance Act for the Elderly, Article 2. (2023).
- National Health Insurance Service. (2023). Long-Term Care Insurance for the Elderly Statistical Yearbook. National Health Insurance Service.
- Korean Board of Geriatric Medicine Committee. (2023). Geriatric Medicine Subspecialist. Euihak Publishing Co.
- Kim, C. O. (2019, October). Frailty and Geriatric Syndrome. Journal of the Korean Association of Internal Medicine Fall Conference, *97*(2), 716–718. Korean Association of Internal Medicine.
- Won, C. W., Yoo, H. J., Yu, S. H., Kim, C. O., Dumlao, L. C. I., Dewiasty, E., ... & Prakash, O. (2013). Lists of geriatric syndromes in the Asian-Pacific geriatric societies. European Geriatric Medicine, 4(5), 335-338.
- Koo, H. J., & Bang, J. S. (2018). Understanding Geriatric Syndrome. Journal of the Korean Society of Pharmaceutical Sciences, 4(1), 41-50.
- National Institute for Korean Medicine Development. (2024). A Study on the Development of a Guidebook for Korean Medicine Home-based Long-term Care Centers. National Institute for Korean Medicine Development.
- Ministry of Health and Welfare. (2025). Pilot Project Guidelines for Long-term Care Home-based Medical Centers (Korean Medicine Clinic). <https://www.mohw.go.kr/board.es?mid=a10501010100&bid=0003&act=view&list_no=1484242&tag=&nPage=1>
- Allen, A., Patrick, H., Ruof, J., Buchberger, B., Varela-Lema, L., Kirschner, J., ... & Guilhaume, C. (2022). Development and pilot test of the registry evaluation and quality standards tool: an information technology–based tool to support and review registries. Value in Health, 25(8), 1390-1398.
- Asher, A. L., Parker, S. L., Rolston, J. D., Selden, N. R., & McGirt, M. J. (2015). Using clinical registries to improve the quality of neurosurgical care. Neurosurgery Clinics of North America, 26(2), 253-63.
- Gliklich, R. E., Dreyer, N. A., & Leavy, M. B. (Eds.). (2014). Registries for evaluating patient outcomes: a user’s guide.
- World Health Organization. (2001). International classification of functioning, disability and health (ICF). WHO.
- Ministry of Health and Welfare. (2018). Notification on Criteria for Determining Long-Term Care Insurance Grade, Article 2 (Method for Calculating Long-Term Care Recognition Score). Ministry of Health and Welfare.
- Fried, L. P., Ferrucci, L., Darer, J., Williamson, J. D., & Anderson, G. (2004). Untangling the concepts of disability, frailty, and comorbidity: implications for improved targeting and care. The journals of Gerontology Series A: Biological sciences and Medical sciences, 59(3), M255-M263.
- World Health Organization. (2025). ICD-11 for mortality and morbidity statistics. https://icd.who.int
- Lee, Y. J.. (2006). Mechanism of Chronic Pain and IMS (Interventional Muscle and Nerve Stimulation). Korean Journal of Family Medicine, 27(5), 341-351.
- Samuelsen, P. J., Svendsen, K., Wilsgaard, T., Stubhaug, A., Nielsen, C. S., & Eggen, A. E. (2016). Persistent analgesic use and the association with chronic pain and other risk factors in the population—a longitudinal study from the Tromsø Study and the Norwegian Prescription Database. European journal of clinical pharmacology, 72(8), 977-985.
- Clark, J. D. (2002). Chronic pain prevalence and analgesic prescribing in a general medical population. Journal of pain and symptom management, 23(2), 131-137.
- Won, C. W., et al. (2002). Development of Korean Activities of Daily Living (K-ADL) and Korean Instrumental Activities of Daily Living (K-IADL) Scales. Journal of the Korean Geriatrics Society, 6(2), 107–120.
- Hyo-Sun You. (2021). Clinical Practice Guideline for Frailty Applicable in Primary Care Setting. Korean Journal of Family Practice, 11(4), 229–236.
- Fried, L. P., Tangen, C. M., Walston, J., Newman, A. B., Hirsch, C., Gottdiener, J., ... & McBurnie, M. A. (2001). Frailty in older adults: evidence for a phenotype. The Journals of Gerontology Series A: Biological Sciences and Medical Sciences, 56(3), M146-M157.
- Williams, A. C. D. C., Davies, H. T. O., & Chadury, Y. (2000). Simple pain rating scales hide complex idiosyncratic meanings. Pain, 85(3), 457-463.
- Mihee Cho., et al. (2024). Explorative Study of Patient Satisfaction and Service Quality for Home-Based Primary Care. Journal of the Korean Geriatrics Society, 44(4), 509-521.
